# PTEN depletion reduces H3K27me3 levels to promote epithelial-to-mesenchymal transition in epithelial colorectal cancer cells

**DOI:** 10.1371/journal.pone.0313769

**Published:** 2024-11-19

**Authors:** Ahmed H. Ghobashi, Jane W. Kimani, Christopher A. Ladaika, Heather M. O’Hagan

**Affiliations:** 1 Genome, Cell, and Developmental Biology Graduate Program, Department of Biology, Indiana University Bloomington, Bloomington, IN, United States of America; 2 Medical Sciences Program, Indiana University School of Medicine, Bloomington, IN, United States of America; 3 Indiana University Melvin and Bren Simon Comprehensive Cancer Center, Indianapolis, IN, United States of America; 4 Department of Medical and Molecular Genetics, Indiana University School of Medicine, Indianapolis, IN, United States of America; Fudan University, CHINA

## Abstract

Epithelial-to-mesenchymal (EMT) transition is one of the best-known examples of tumor cell plasticity. EMT enhances cancer cell metastasis, which is the main cause of colorectal cancer (CRC)-related mortality. Therefore, understanding underlying molecular mechanisms contributing to the EMT process is crucial to finding druggable targets and more effective therapeutic approaches in CRC. In this study, we demonstrated that phosphatase and tensin homolog (PTEN) knockdown (KD) induces EMT in epithelial CRC, likely through the activation of AKT. PTEN KD modulated chromatin accessibility and reprogrammed gene transcription to mediate EMT in epithelial CRC cells. Active AKT can phosphorylate enhancer of zeste homolog 2 (EZH2) on serine 21, which switches EZH2 from a transcriptional repressor to an activator. Interestingly, PTEN KD reduced the global levels of trimethylation of histone 3 at lysine 27(H3K27me3) in an EZH2-phosphorylation-dependent manner. Additionally, EZH2 phosphorylation at serine 21 reduced the interaction of EZH2 with another polycomb repressive complex 2 (PRC2) component, suppressor of zeste 12 (SUZ12), suggesting that the reduced H3K27me3 levels in PTEN KD cells were due to a disruption of the PRC2 complex. Overall, we demonstrated that PTEN KD modulates changes in gene expression to induce the EMT process in epithelial CRC cells by phosphorylating EZH2 and activates transcription factors such as activator protein 1 (AP1).

## Introduction

Cellular plasticity allows cells to adopt different phenotypic characteristics during development and proliferation [1]. One of the most common examples of cellular plasticity is the epithelial-to-mesenchymal transition (EMT) [1, 2]. During EMT, epithelial cells switch to spindle-like mesenchymal cells and acquire distinct mesenchymal features such as increased cell migration and invasion. EMT plays an essential role in normal physiological conditions, including wound healing and embryogenesis [2]. EMT occurs also during pathological conditions such as cancer progression and metastasis. EMT increases the invasiveness and metastatic behavior of cancer cells [2].

The EMT process is regulated by the expression of EMT-transcription factors (EMT-TFs) such as TWIST1/2, SNAI1/2, and ZEB1/2 [3]. EMT-TFs are essential to induce the expression of mesenchymal-related genes [4]. The expression of EMT-TFs is regulated by different signaling pathways such as NOTCH, FGF, PI3K/AKT, and TGFβ [5]. In addition to signaling pathways, epigenetic modifications such as histone H3 lysine 27 trimethylation (H3K27me3) are known to regulate the expression of EMT-TFs to influence the EMT process [6, 7].

Polycomb repressive complex 2 (PRC2) deposits the repressive chromatin mark H3K27me3 [8, 9]. H3K27me3 plays an essential role in maintaining cellular lineage commitment during differentiation and upholding cell identity through repressing gene expression [10, 11]. Enhancer of zeste homolog 2 (EZH2) is a lysine methyltransferase and acts as the catalytic subunit for PRC2. Other core components of PRC2 include the suppressor of zeste 12 (SUZ12) and Embryonic Ectoderm Development (EED) [9]. While SUZ12 is essential to maintain PRC2 complex integrity [12, 13], EED enhances the allosteric activity of PRC2 [14]. Therefore, SUZ12 and EED are essential for PRC2 to be functional.

Several studies have suggested that signaling pathways such as PI3K/AKT modulate the function of EZH2 [15–17]. Phosphorylation of EZH2 at serine 21 by AKT has been shown to switch the function of EZH2 to act as a transcriptional activator rather than a transcriptional repressor [16, 17]. Additionally, phosphorylation of EZH2 at serine 21 reduces the global levels of H3K27me3 in certain contexts [18]. For example, AKT-mediated EZH2 phosphorylation reduced EZH2 affinity for histones resulting in a reduction in H3K27me3 levels in breast cancer [18]. We previously showed that AKT-mediated EZH2 phosphorylation induced EZH2 to interact with and methylate β-catenin in mesenchymal colorectal cancer (CRC) cells such as SW480 [15]. However, in these mesenchymal CRC cells, AKT-mediated EZH2 phosphorylation did not alter global H3K27me3 levels.

Consensus molecular subtyping (CMS) classifies CRC cells into four classes based on their gene expression profiles [19]. CMS3 and CMS4 are characterized as epithelial and mesenchymal, respectively. Additionally, CMS4 CRC is associated with poor prognosis and therapy resistance [19]. In our previous work, we studied the AKT/EZH2 axis mainly in the mesenchymal SW480 cell line, which is classified as CMS4 [20]. Because our previous work showed that activation of AKT increased EMT gene expression in CMS4 SW480 cells, in this study, we explored how AKT activation alters EMT in CMS3 epithelial CRC cell lines.

Loss of phosphatase and tensin homolog (PTEN) increases the activity of the PI3K/AKT pathway and occurs in approximately 30% of CRCs [21]. PTEN loss and activation of PI3K/AKT have been demonstrated to positively regulate EMT [22–24]. For example, loss of PTEN has been reported to increase Snail nuclear translocation to induce EMT in lung cancer [24]. We have previously shown that activation of AKT stabilizes Snail protein to mediate EMT in CRC cells [25]. Here, we demonstrated that, following activation of the PI3K/AKT pathway via PTEN knockdown (KD), epithelial CRC cells transitioned to mesenchymal spindle-like cells. PTEN KD increased chromatin accessibility and induced gene transcription reprogramming to push epithelial CMS3 cells toward mesenchymal CMS4 cells. Additionally, PTEN KD induced a reduction in H3K27me3 levels that is dependent on EZH2 phosphorylation at serine 21. The phosphorylation of EZH2 reduced the interaction of EZH2 with another PRC2 component, SUZ12. Furthermore, PTEN KD increased the transcriptional activity of Activator Protein 1 (AP1), which has been shown to induce EMT-TF expression in epithelial cells to mediate EMT [26]. Our work suggests that AKT-mediated EZH2 phosphorylation reduces the integrity of the PRC2 complex in epithelial CRC cells, attenuating the catalytic activity of PRC2 and enhancing the transcriptional activity of AP1 resulting in increased expression of mesenchymal-related genes.

## Results

### PTEN KD enhances epithelial CRC cell migration

**Fig 1:**
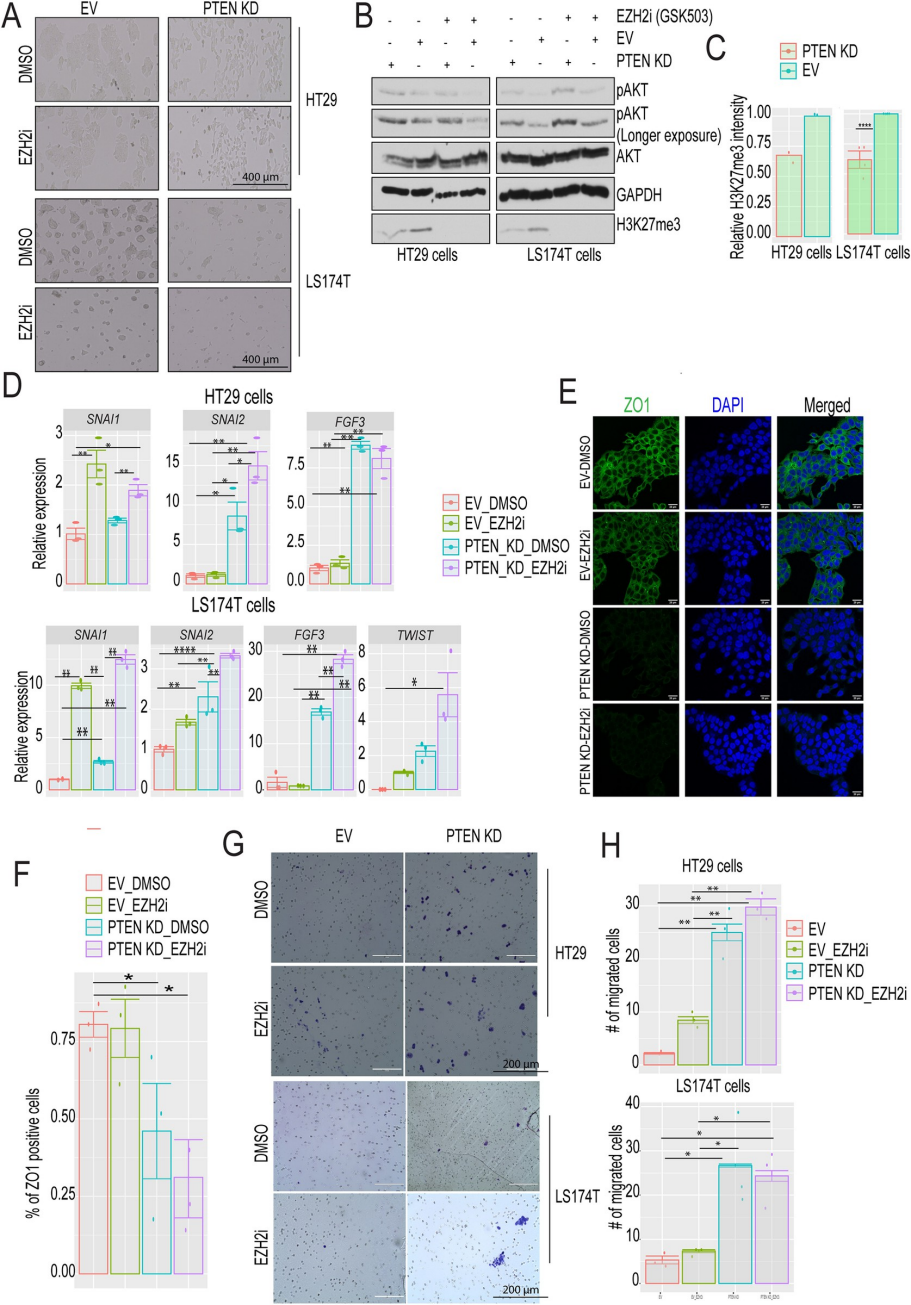
PTEN KD induces EMT in epithelial CRC cells. **(A)** Brightfield images of empty vector (EV) and PTEN knockdown (KD) HT29 and LS174T cells. Cells were treated with DMSO or EZH2 inhibitor (EZH2i, GSK503, 2 μM, 72 h). Cells were starved in media lacking serum for 24 hours prior to imaging. Representative images of N = 3 independent experiments. Scale bar = 400μm. (**B)** Western blots of total cell lysates prepared from EV and PTEN KD HT29 and LS174T cells treated as in (A). Cells were starved in media lacking serum for 24 hours prior to cell lysate preparation. EZH2i treated EV and PTEN KD cells act as a control for decreased H3K27me3 levels. **(C)** H3K27me3 band quantification for untreated EV and PTEN KD in HT29 and LS174T cells in (B). Relative H3K27me3 band intensity measured by imageJ and normalized to the housekeeping gene GAPDH. N = 2 HT29, N = 4 LS174T biological replicates. **(D)** Gene expression of the indicated genes by RT-qPCR in HT29 and LS174T cells treated as in (A). The relative expression levels of indicated genes were measured using the Delta Delta Cq method. Cq values were normalized to the housekeeping gene *RHOA* expression and then to EV_DMSO. Cells were starved in media lacking serum for 24 hours prior to RNA extraction. Results are represented as the means of 3 biological replicates +/- SEM. **(E)** Immunofluorescence of EV and PTEN KD HT29 cells cultured on coverslips in a 6-well plate and treated as in (A). Cells were starved in media lacking serum for 24 hours prior to staining. Scale bar = 20μm. **(F)** Quantification of ZO1 positive cells in (E). Results are represented as the mean of 3 biological replicates +/- SEM. **(G)** EV and PTEN KD HT29 and LS174T cells were treated with DMSO or 2 μM EZH2 inhibitor (EZH2i, GSK-503) for 72 h followed by plating cells in the upper chamber of a transwell insert. Brightfield images of crystal violet–stained migrated cells were taken after 48 h. Scale bar = 200 μm. **(H)** Quantification of migration normalized to migration counts for EV-DMSO cells. Migration inserts were randomized before manual quantification and the outer 5% of the inserts were not included during quantification to reduce edge-effect bias. Results are represented as the mean of 3 biological replicates +/- SEM. Significance was determined by one-way ANOVA with the Tukey multiple comparisons test. All significant comparisons are shown. * P ≤ 0.05, ** P ≤ 0.01, *** P ≤ 0.001, **** P ≤ 0.0001.

We have previously shown that activation of AKT via PTEN KD in mesenchymal SW480 cells increased cell migration. Additionally, treating PTEN KD SW480 cells with an EZH2 inhibitor (GSK 503) further increased cell migration [15]. To extend our previous findings, we tested the effect of PTEN KD and EZH2 inhibition on epithelial CMS3 CRC cells. We used HT29 and LS174T cells throughout this study because they are characterized as epithelial CMS3 cells [20]. PTEN KD HT29 and LS174T cells displayed a distinct morphologic phenotype compared to empty vector (EV)-DMSO cells. PTEN KD-DMSO cells formed looser colonies with a more elongated spindle-like cell morphology typically associated with the mesenchymal phenotype (Fig 1A). Treating EV HT29 and LS174T cells with an EZH2 inhibitor for 4 days did not affect cell morphology (Fig 1A). As expected, the EZH2 inhibitor drastically reduced the level of H3K27me3 (Fig 1B and 1C). Additionally, PTEN KD increased levels of phosphorylated AKT in HT29 and LS174T cells. Interestingly, PTEN KD alone reduced H3K27me3 levels in HT29 and LS174T cells (Fig 1B and 1C). Furthermore, PTEN KD and/or EZH2 inhibition increased the expression of some EMT-related genes in HT29 and LS174T cells (Fig 1D and S1A Fig). Specifically, inhibiting EZH2 activity using GSK503 or Tazemetostat, an FDA-approved EZH2 inhibitor, in EV HT29 cells significantly up-regulated the expression of *SNAI1* with no effect on *SNAI2* or *FGF3*. While PTEN KD alone increased the expression of the *SNAI2* and *FGF3* with no effect on *SNAI1* relative to EV-DMSO treated cells, inhibiting EZH2 activity in PTEN KD HT29 cells significantly increased the expression of all three genes relative to the EV control (Fig 1D and S1A Fig). Both EZH2 inhibitors and PTEN KD also increased the expression of a subset of the EMT-related genes in LS174T. Inhibiting EZH2 activity in PTEN KD LS174T resulted in further upregulation of these genes (Fig 1D and S1A Fig). The loose colony phenotype induced by PTEN KD (Fig 1A) led us to test for the presence of ZO1 by immunofluorescence. ZO1 is important for cell junction and cell adhesion [27]. ZO1 was localized in the cytoplasm in both EV-DMSO and EV-EZH2i HT29 cells. PTEN KD-DMSO and PTEN KD-EZH2i reduced ZO1 staining relative to EV cells, with the reduction being significant in PTEN KD-EZH2i cells (Fig 1E and 1F). Consistent with the enhanced migratory phenotype of mesenchymal cells, PTEN KD increased both HT29 and LS174T cell migration. In contrast, EZH2 inhibitor treatment had little or no effect on cell migration (Fig 1G and 1H). Additionally, PTEN KD reduced colony formation in HT29 and LS174T compared to EV cells (S1B and S1C Fig). These results suggest that PTEN KD switches epithelial CRC cells toward the mesenchymal lineage.

### PTEN KD induces transcriptional reprogramming to mediate an epithelial-to-mesenchymal switch

**Fig 2:**
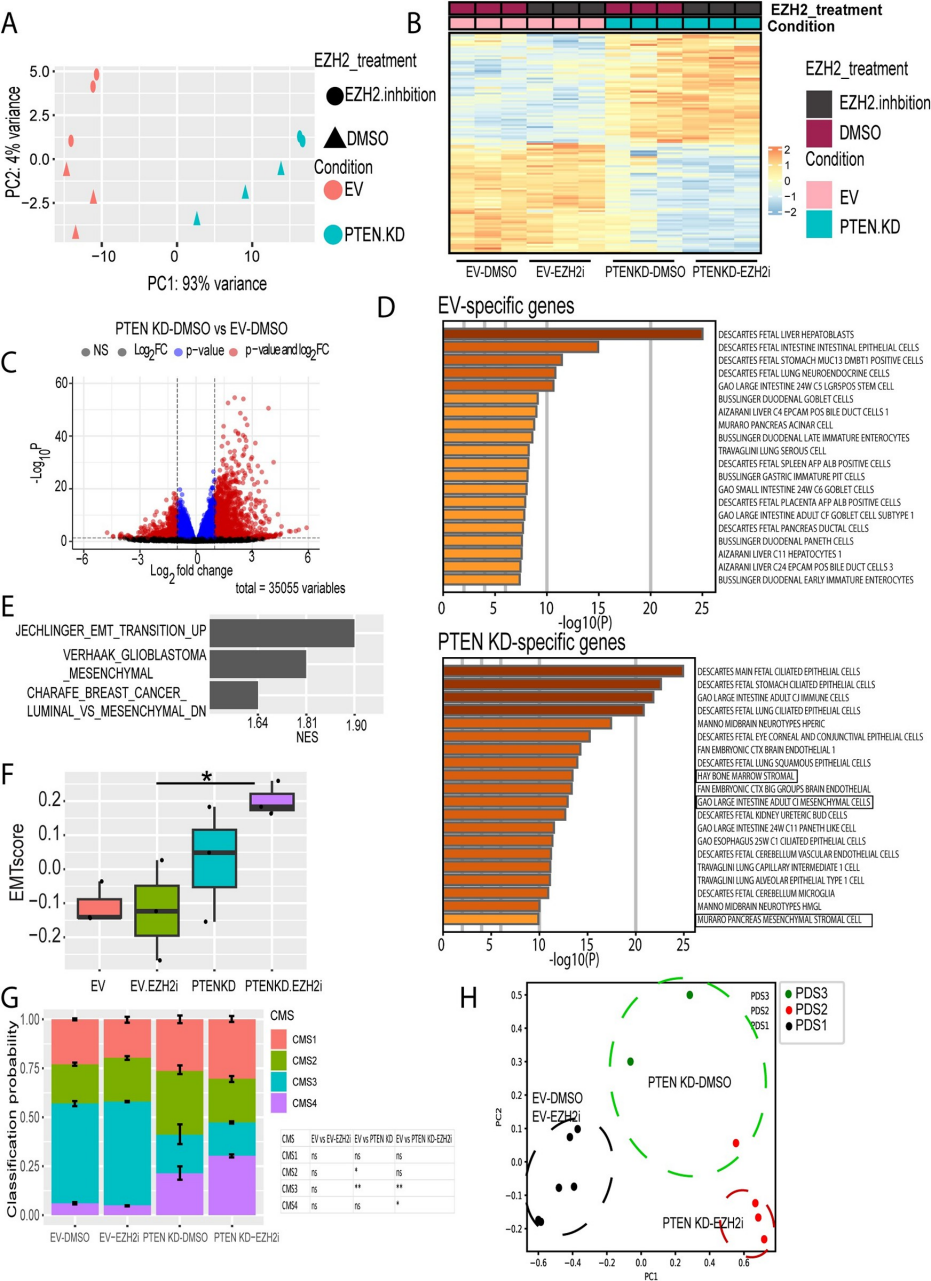
PTEN KD mediates transcriptional reprogramming toward a mesenchymal lineage. **(A)** PCA plot of RNA-seq samples showing the variance between empty vector (EV)-DMSO, EV-EZH2i, PTEN knockdown (KD)-DMSO, and PTEN KD-EZH2i. Cells were treated with DMSO or EZH2 inhibitor (EZH2i, GSK503, 2 μM, 72 h). **(B)** Heatmap for significantly differentially expressed genes (DEGs, (|log 2FC|>1; p < 0.05)) in PTEN KD-DMSO versus EV-DMSO. Heatmap displays the gene expression profiles of the indicated samples. Hierarchical unsupervised clustering was performed to group genes and samples based on their expression patterns. The color intensity represents the z-score of gene expression. Higher z-scores (red) indicate higher expression levels, while lower z-scores (blue) indicate lower expression levels. **(C)** Volcanoplot for DEGs in PTEN KD-DMSO versus EV-DMSO from RNA-seq data in A. Dashed lines represent Log2 Fold change > |1| and p-adj value <0.05. Red dots indicate genes significantly up or down regulated compared to EV with Log2 Fold change > |1|, blue dots indicate genes Log2 Fold change < |1|. Black dots indicate nonsignificant gene expression. **(D)** Bar plot for cell signatures generated by Metascape for PTEN KD-down regulated genes relative to EV (top) and PTEN KD-up regulated genes relative to EV (bottom). Genesets related to mesenchymal cells are boxed. **(E)** Barplot for the GSEA of metastasis datasets for significant PTEN KD-up regulated genes relative to EV**. (F)** Box plot for EMT score for the indicated RNA-seq samples. EMT score nearer to +1.0 is more mesenchymal, whereas EMT score nearer to 1.0 is more epithelial. Results are represented as the mean of 3 biological replicates +/- SEM. **(G)** Stacked bar plot for CMS classification probability for EV-DMSO, EV-EZH2i, PTEN KD-DMSO, and PTEN KD-EZH2i RNA-seq samples. Results are represented as the mean of 3 biological replicates +/- SEM. **(H)** PCA plot for K-mean clustered (K = 3) samples showing PDS classification. Dash lines were drawn manually to encircle EV-DMSO/EV-EZH2i (Black), PTEN KD-DMSO (Green), and PTEN KD-EZH2i (red) samples. Significance was determined by one-way ANOVA with the Tukey multiple comparisons test. All significant comparisons are shown. * P ≤ 0.05, ** P ≤ 0.01. ns-not significant.

To determine the impact of AKT activation via PTEN KD and EZH2 activity on transcription, we performed RNA-seq with and without EZH2 inhibition in EV and PTEN KD HT29 cells. As indicated by the PCA plot, most of the variance between the samples was from the condition (EV and PTEN KD, PC1; 93% variance, Fig 2A). Significantly differentially expressed genes (DEGs, (|log 2FC|>1; p < 0.05) in PTEN KD-DMSO versus EV-DMSO were determined (Fig 2B). PTEN KD resulted in 1145 upregulated and 412 downregulated genes compared to EV-DMSO (|log 2FC|>1; p < 0.05; Fig 2C). EZH2 inhibition did not alter the expression pattern induced by PTEN KD in HT29 cells (Fig 2B) with only 16 up-regulated and 13 down-regulated genes in PTEN KD-EZH2i compared to PTEN KD-DMSO (|log 2FC|>1; p < 0.05, S2A Fig). Furthermore, as demonstrated in the PCA plot, the EZH2 inhibitor had a small effect on the variance between the samples (PC2; 4% variance, Fig 2A). Inhibiting EZH2 in EV resulted in 106 upregulated and 28 downregulated genes compared to EV-DMSO (|log 2FC|>1; p < 0.05; S2B Fig). These results show that, in contrast to our previous findings in mesenchymal CMS4 SW480 CRC cells [15], inhibiting EZH2 activity in epithelial CMS3 HT29 cells had a minor effect on gene expression compared to PTEN KD.

To gain more insight from the transcriptional data, we performed gene ontology analysis (GO) on the lists of DEGs. GO analysis for up-regulated genes in PTEN KD-DMSO and PTEN KD-EZH2i compared to EV-DMSO showed enrichment for pathways related to immune response, interferon signaling, cell motility, cell adhesion, and morphogenesis (S2C and S2D Fig). GO analysis for down-regulated genes in PTEN KD-DMSO and PTEN KD-EZH2i versus EV-DMSO indicated enrichment of pathways related to metabolic processes such as amino acid metabolism and hormone regulation (S2E and S2F Fig). Furthermore, cell signature analysis for down-regulated genes in PTEN KD-DMSO versus EV-DMSO (EV-specific genes) showed enrichment for epithelial cells such as hepatocytes, enterocytes, and intestinal epithelial cells (Fig 2D, EV-specific genes). The cell signature analysis on genes upregulated in PTEN KD-DMSO versus EV-DMSO included significantly enriched cell signatures for intestinal mesenchymal and stromal cells in addition to epithelial cells (Fig 2D, PTEN KD-specific genes).

As we had seen phenotypic differences related to EMT with PTEN KD, we further examined our RNA-seq data specifically for gene expression changes related to EMT. Gene set enrichment analysis (GSEA) for upregulated genes in PTEN KD-DMSO versus EV-DMSO showed significant enrichment for different EMT datasets (Fig 2E). Additionally, we calculated EMT scores, which range between 1 (fully mesenchymal) and -1 (fully epithelial), for our samples. PTEN KD increased the EMT score with a further increase in the PTEN KD-EZH2i samples (Fig 2F). CMS classification showed that EV-DMSO HT29 cells had higher probabilities for CMS3 than the other CMS classifications (Fig 2G), which is consistent with HT29 cells previously being classified as CMS3 [20]. Additionally, inhibiting EZH2 activity in EV HT29 had little effect on CMS classification compared to EV-DMSO (Fig 2G). Interestingly, PTEN KD-DMSO and PTEN KD-EZH2i showed a significant reduction in CMS3 probability with a significant increase in CMS4 probability in PTEN KD-EZH2i compared to EV-DMSO (Fig 2G). To further confirm our CMS results, we used pathway-derived subtyping (PDS) as another method to classify our samples [28]. While PDS1 is characterized by being epithelial, PDS2 and PDS3 are characterized as stromal and intermediate, respectively [28]. Intriguingly, PDS predicted all PTEN KD-EZH2i samples and one sample of PTEN KD-DMSO as PDS2, and two PTEN KD-DMSO samples as PDS3 (Fig 2H). All samples from both EV-DMSO and EV-EZH2i were predicted as PDS1 (Fig 2H). These data suggest that inhibiting the catalytic activity of EZH2 alone has little effect on gene expression whereas AKT activation through PTEN KD induces transcriptional reprogramming to mediate a transition to a more mesenchymal phenotype in epithelial CMS3 CRC.

### PTEN KD increases chromatin accessibility in CRC epithelial cells

**Fig 3:**
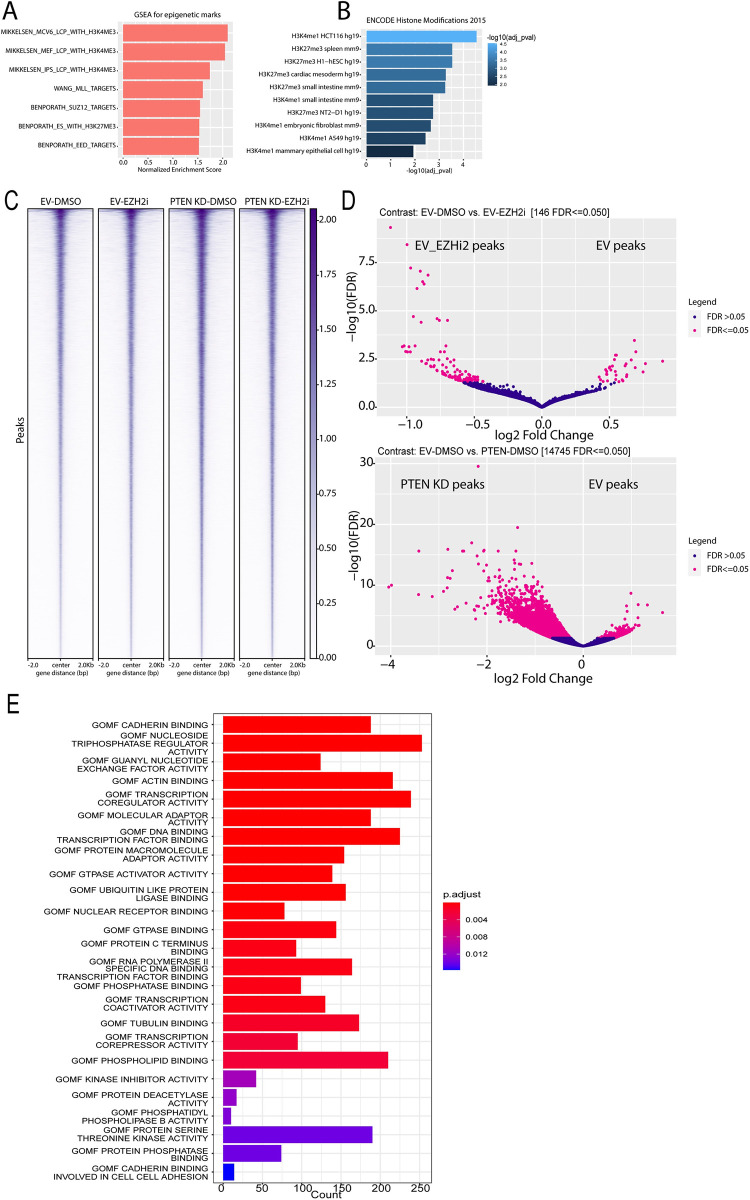
PTEN KD increases chromatin accessibility. **(A)** Barplot of normalized enrichment scores for genesets with significant enrichment by GSEA using the epigenetic geneset dataset and genes significantly upregulated in PTEN knockdown (KD) relative to empty vector (EV) from the RNA-seq data in Fig 2 (|log 2FC|>1; p < 0.05). **(B)** Barplot of adjusted p-values by GSEA using genesets from the ENCODE Histone Modification 2015 and significantly PTEN KD-up regulated genes (|log 2FC|>1; p < 0.05). **(C)** Metagenomic heatmap for ATAC-seq prepared from EV and PTEN KD HT29 cells treated with DMSO or 2 μM EZH2 inhibitor (EZH2i, GSK-503) for 72 hrs. The heatmap displays the chromatin accessibility profiles of the indicated samples. Peaks were combined across all samples. The color legend represents the magnitude of each peak. The higher the number the higher the chromatin accessibility. **(D)** Volcanoplot for differentially accessible peaks (|log 2FC|>0.5; False discovery rate (FDR) < = 0.05) in EV-DMSO versus EV-EZH2i. (Top) and EV-DMSO versus PTEN KD-DMSO (bottom). Red dots indicate peaks with significantly increased or decreased accessibility with Log2 FC > |0.5|. Blue dots indicate peaks with nonsignificant changes in accessibility **(E)** Gene ontology bar plot for genes with a significant increase in chromatin accessibility in PTEN KD-DMSO versus EV-DMSO. X-axis represents the number of genes in both datasets. Legend color represents the adjusted p value.

PDS1 and PDS3 are characterized by low and high expression of PRC2 target genes in CRC cells, respectively [28]. PTEN KD reduced the H3K27me3 levels in HT29 and LS174T (Fig 1B and 1C). To further explore the connection between PTEN KD-regulated genes and H3K27me3, we performed GSEA analysis using up-regulated genes in PTEN KD-DMSO versus EV-DMSO from our RNA-seq data (log 2FC>1; p < 0.05) and epigenetic mark datasets. This analysis showed significant enrichment for genes regulated by H3K4me3, H3K27me3, and PRC2 components, SUZ12 and EED (Fig 3A). Additionally, analysis using the Encode Histone Modifications 2015 database for the same gene list showed significant enrichment for H3K4me1 and H3K27me3 (Fig 3B). However, PTEN KD did not alter the total levels of H3K4me3 in HT29 cells (S3A Fig). These data suggest that PTEN KD might induce transcriptional reprogramming via reducing H3K27me3 levels to mediate the mesenchymal transition in the epithelial CMS3 CRC cells.

H3K27me3 is known to induce chromatin compaction [29]. Therefore, the finding that PTEN KD reduced the level of H3K27me3 caused us to investigate how PTEN KD altered chromatin accessibility. Our Assay for Transposase-Accessible Chromatin with high-throughput sequencing (ATAC-seq) data showed that PTEN KD increased chromatin accessibility compared to EV-DMSO HT29 cells (replicate1; Fig 3C and repicate2; S3B Fig). Although EZH2 inhibition reduced the global levels of H3K27me3 (Fig 1B and 1C), EZH2 inhibition had a limited effect on chromatin accessibility with 100 increased and 23 reduced differentially accessible peaks compared to EV-DMSO (|log 2FC|>0.5; FDR < = 0.05; Fig 3D, top). Interestingly, PTEN KD-DMSO cells predominantly had increased chromatin accessibility with 10,164 increased and 211 reduced differential accessible peaks compared to EV-DMSO (|log 2FC|>0.5; FDR < = 0.05; Fig 3D, bottom). Peaks were annotated to the nearest genes (+/-3 kb) to gain more insights from our ATAC-seq data. Genes annotated to EV-specific peaks (204 genes) showed significant enrichment for pathways related to glycosylation (S3C Fig). Genes annotated to PTEN KD-specific peaks, 6899 genes, showed significant pathways related to cell adhesion, cadherin binding, transcription factor DNA binding, RNAPII DNA binding, and transcription coactivator activity (Fig 3E) indicating that the open chromatin in PTEN KD is accompanied by active transcription. These data also suggest that inhibiting EZH2 activity alone has a much smaller effect on chromatin accessibility compared to PTEN KD.

### PTEN KD increases chromatin accessibility at EMT-related genes

To understand the relationship between chromatin accessibility and gene expression, we performed Pearson’s correlation analysis between differentially accessible genes (DAGs) in ATAC-seq data and DEGs in RNA-seq data in PTEN KD-DMSO versus EV-DMSO. There was a significant positive correlation (0.236, p-value < 2.2e^-16^, S4A Fig) between DAGs and DEGs suggesting that chromatin accessibility induced by PTEN KD is positively associated with gene expression. By integrating genes with increased chromatin accessibility in PTEN KD-DMSO vs EV-DMSO (log 2FC>0.5; p < 0.05) with up-regulated genes expressed in PTEN KD-DMSO vs EV-DMSO (log 2FC>0.5; p < 0.05) we identified 1018 genes that were present in both datasets (S4B Fig). Some of these genes are known to promote EMT such as *TNC*, *ANO1*, *TMEM40*, and *ITGB4* (S4C Fig). We performed RT-qPCR to confirm the effect of PTEN KD on the expression of some of these genes. PTEN KD significantly increased the expression of *ANO1* and *ITGB4* compared to EV (S4D Fig). We also assayed the expression of other known EMT related genes and determined that PTEN KD did not alter the expression of E-cadherin (*CDH1*) but did cause a trend toward increased N-cadherin (*CDH2*) expression (S4D Fig). Furthermore, GO analysis of the 1018 common genes resulted in identification of enriched pathways involved in morphogenesis, response to wounding, and embryonic development (S4E Fig).

**Fig 4:**
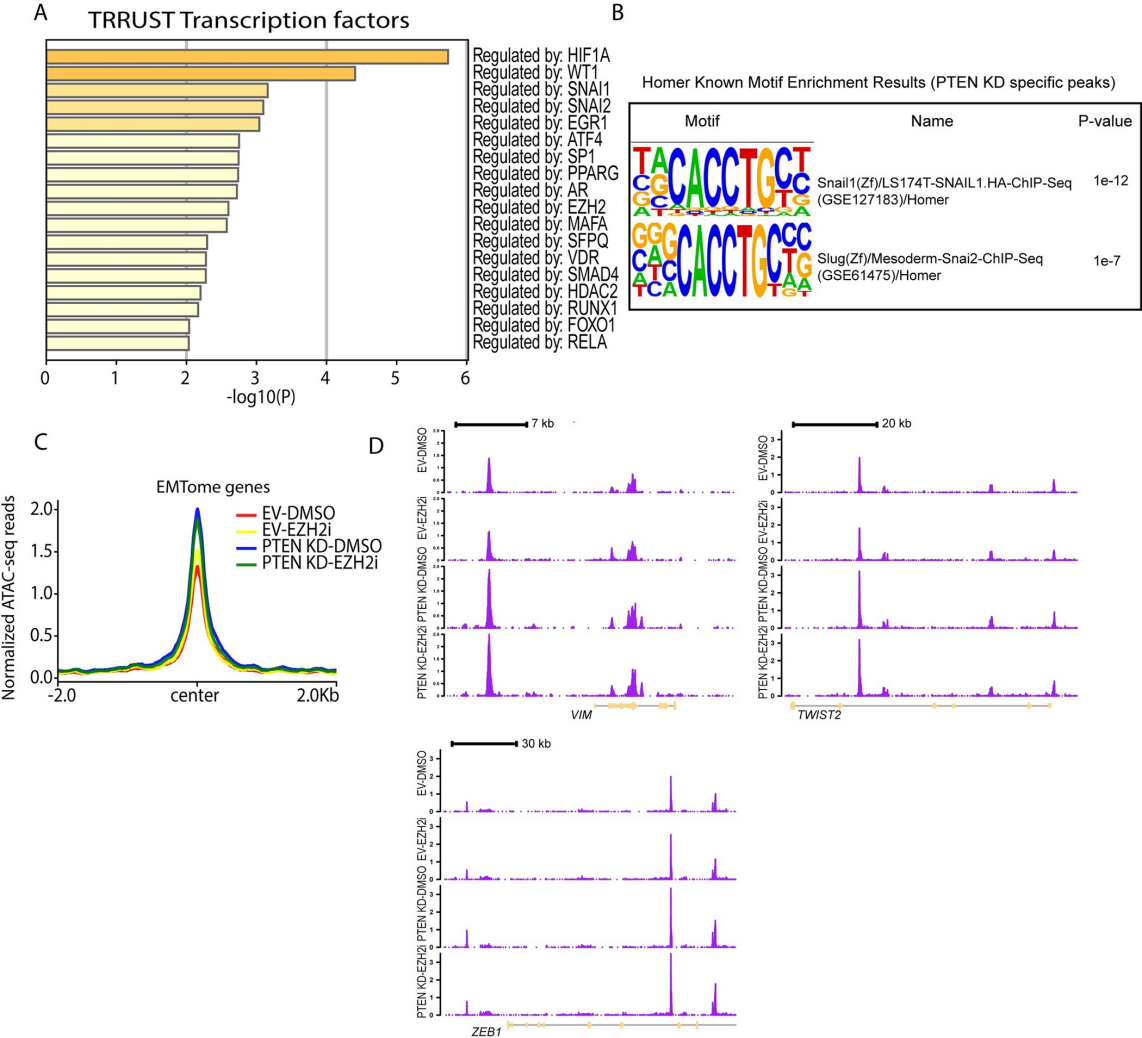
Activation of AKT increases chromatin accessibility at EMT genes. **(A)** Bar plot of the p-values generated by Metascape using the TRUUST transcription factors genesets and genes with increased chromatin accessibility in PTEN knockdown (KD)-DMSO compared to empty vector (EV)-DMSO from ATAC-seq data. **(B)** Motif enrichment in PTEN KD specific peaks in ATAC-seq data calculated using Homer. **(C)** Count per million (CPM) normalized ATAC-seq reads for EV-DMSO, EV-EZH2i, PTEN KD-DMSO, and PTEN KD-EZH2i at EMTome genes. EMTome genes were obtained from the EMTome database (https://www.emtome.org/) **(D)** ATAC-seq gene tracks for indicated genes in EV-DMSO, EV-EZH2i, PTEN KD-DMSO, and PTEN KD-EZH2i. Y-axis represents the signal intensity measured as normalized ATAC-seq reads.

To gain more insights into possible transcription factors that could regulate the DAGs, we performed the TRRUST transcription factors analysis using Metascape [30]. TRRUST transcription factors database provides insight into different transcription factor-gene target relationships [31]. Genes with increased chromatin accessibility in PTEN KD-DMSO versus EV-DMSO (log 2FC>0.5, p<0.05) showed significant enrichment for transcription factors HIF1A, SNAI1, SNAI2, and EZH2 (Fig 4A). Additionally, Homer motif analysis for increased accessible regions in PTEN KD-DMSO versus EV-DMSO showed significant enrichment for SNAI1 and SNAI2 motifs (Fig 4B). SNAI1 and SNAI2 are known EMT-TFs that are involved in the EMT process [4]. To get a broader view of the chromatin accessibility at EMT genes in our samples, we used the EMTome dataset that contains 1153 genes involved in metastasis [32]. PTEN KD-DMSO and PTEN KD-EZH2i showed increased chromatin accessibility at EMT genes compared to EV-DMSO and EV-EZH2i (Fig 4C) as exemplified by *TNC*, *ANO1*, and *ITGB4* (S4C Fig) and *VIM*, *TWIST2*, and *ZEB1* genes (Fig 4D). These results demonstrate that PTEN KD increases the chromatin accessibility at EMT-related genes, which is associated with the PTEN KD-induced transcriptional reprogramming to mediate EMT.

### AKT-mediated EZH2 phosphorylation contributes to EMT

**Fig 5:**
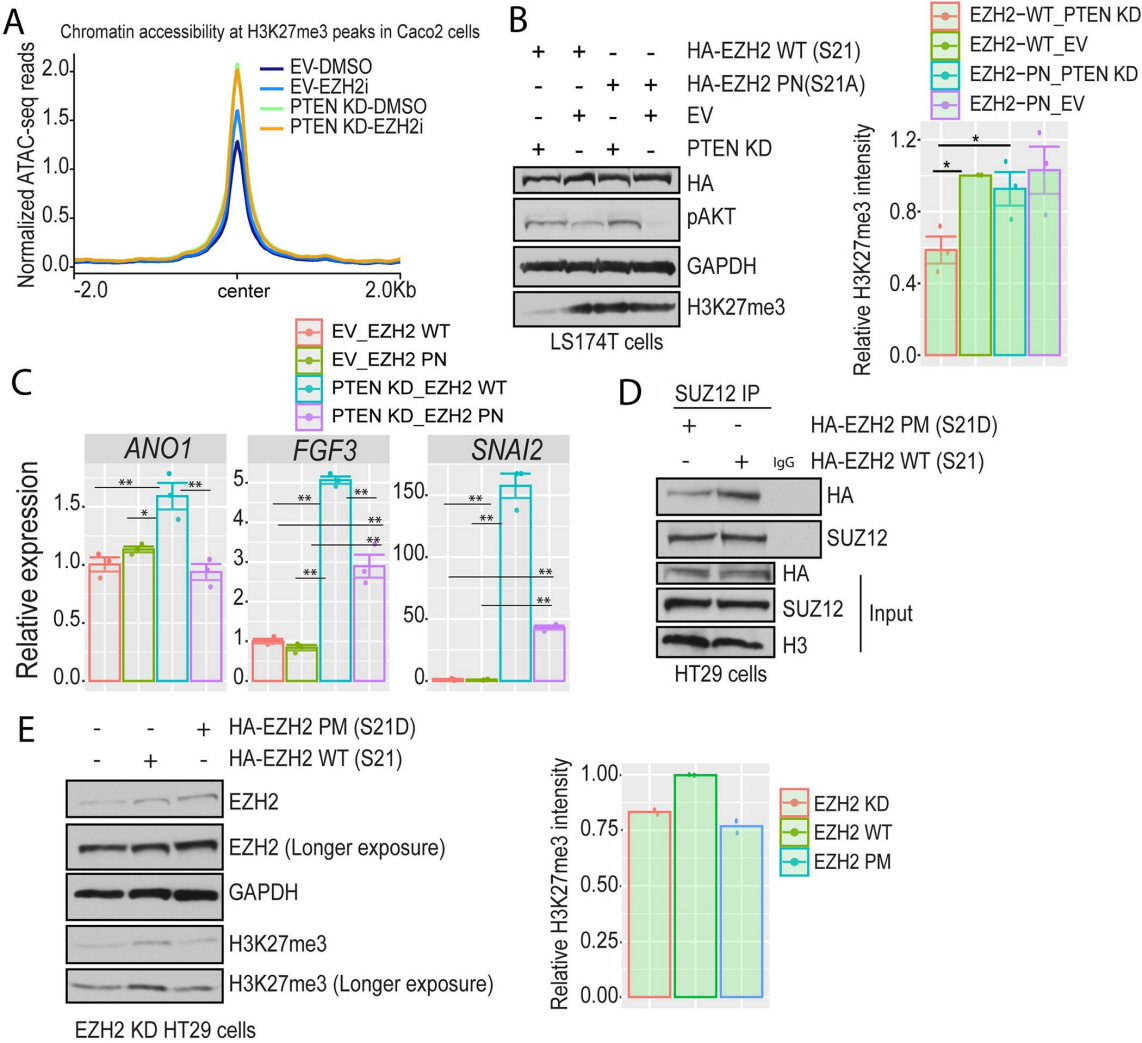
Phosphorylation of EZH2 disrupts PRC2 integrity. **(A)** Count per million (CPM) normalized ATAC-seq reads for empty vector (EV)-DMSO, EV-EZH2i, PTEN knockdown (KD)-DMSO, and PTEN KD-EZH2i at H3K27me3 mint-ChIP-seq peaks generated from Caco2 cells. **(B)** Western blots of total cell lysates prepared from EV and PTEN KD LS174T cells transfected with HA-EZH2 wildtype (WT) and HA-EZH2-phospho null (PN) (Left). Cells were starved in media lacking serum for 24 hours prior to cell lysis. Relative H3K27me3 band intensity for the indicated samples measured by imageJ and normalized to the housekeeping gene GAPDH (Right). Results are represented as the mean of 3 biological replicates +/- SEM. Significance was determined by one-way ANOVA with the Tukey multiple comparisons test. All significant comparisons are shown. * P ≤ 0.05. **(C)** Gene expression of the indicated genes by RT-qPCR in PTEN KD and EV HT29 cells transfected with EZH2 WT or EZH2 PN. Cells were starved in media lacking serum for 24 hours prior to RNA extraction. The relative expression levels of indicated genes were measured using the Delta Delta Cq method. Cq values were normalized to the housekeeping gene *RHOA* expression and then to EV_EZH2 WT. Results are represented as the mean of 3 biological replicates +/- SEM. Significance was determined by one-way ANOVA with the Tukey multiple comparisons test. All significant comparisons are shown. * P ≤ 0.05, ** P ≤ 0.01. **(D)** SUZ12 IP in HT29 cells. HT29 cells were plated followed by transfection with HA-EZH2 WT or HA-EZH2 phosphomimetic (PM) followed by nuclear protein interaction. Nuclear protein lysate was used for the IPs. IP with IgG serves as a negative control. Input is nuclear lysates used for IP. **(E)** Western blots of total cell lysates prepared from EZH2 KD HT29 cells transfected with HA-EZH2 WT or HA-EZH2-PM. EZH2 KD HT29 cells were plated followed by mock transfection or transfection with HA-EZH2 WT or HA-EZH2-PN (Left). Relative H3K27me3 band intensity for the indicated samples measured by imageJ and normalized to the housekeeping gene GAPDH (Right). Results are represented as the mean of 2 biological replicates.

We showed previously that PTEN KD reduces the global levels of H3K27me3 (Fig 1B and 1C). To test if PTEN KD alters chromatin accessibility at H3K27me3 sites in CRC, we used H3K27me3 (multiplex/low input) Mint-ChIP-seq data from Caco2 CRC cells [33]. We found that inhibiting EZH2 activity increased chromatin accessibility at H3K27me3 sites compared to EV-DMSO (Fig 5A). PTEN KD-DMSO and PTEN KD-EZH2i further increased the chromatin accessibility at sites normally marked by H3K27me3 compared to EV-DMSO and EV-EZH2i (Fig 5A). In our previous publication, we demonstrated that activation of AKT induces EZH2 phosphorylation at serine 21 in CRC [15]. To test if PTEN KD-induced reduction of H3K27me3 level was dependent on EZH2 phosphorylation at serine 21, we used our EZH2 (S21A) phospho-null (PN) mutant. Interestingly, EZH2 PN expression diminished the effect of the PTEN KD-induced reduction in H3K27me3 in LS174T (Fig 5B). Additionally, PTEN KD in HT29 cells transfected with EZH2 WT (PTEN KD-EZH2 WT) showed significant upregulation of a subset of EMT-related genes, *ANO1*, *FGF3*, and *SNAI2* compared to EV-EZH2 WT (Fig 5C). Interestingly, transfecting PTEN KD HT29 cells with EZH2 PN abrogated the effect of PTEN KD-induced upregulation of *ANO1* (Fig 5C). Additionally, PTEN KD-EZH2 PN showed significant down-regulation of *FGF3* and *SNAI2* compared to PTEN KD-EZH2 WT (Fig 5C). These results demonstrate that AKT-mediated EZH2 phosphorylation at serine 21 contributes to the expression of EMT genes induced by PTEN KD.

To gain more insights into the mechanism for how PTEN KD reduces H3K27me3, we tested if pS21-EZH2 reduces the integrity of the PRC2 complex. SUZ12 had reduced interaction with EZH2 S21D phospho-mimetic (PM) compared to EZH2 wild-type (WT) in HT29 and LS174T (Fig 5D and S5A Fig). Additionally, EZH2 KD HT29 and LS174T cells rescued with EZH2 PM did not restore H3K27me3 levels unlike EZH2 KD cells rescued with EZH2 WT (Fig 5E and S5B Fig). Consistent with our previous finding where EZH2 phosphorylation induced EZH2’s interaction with RNAPII in mesenchymal CRC cells [15], PTEN KD induced EZH2 to interact with RNAPII in epithelial CRC LS174T cells (S5C Fig). These data suggest that phosphorylation of EZH2 at S21 increases EZH2 interaction with RNAPII and reduces the interaction of EZH2 with SUZ12, hence disrupting PRC2 complex integrity.

### PTEN KD activates AP1 transcriptional activity

**Fig 6:**
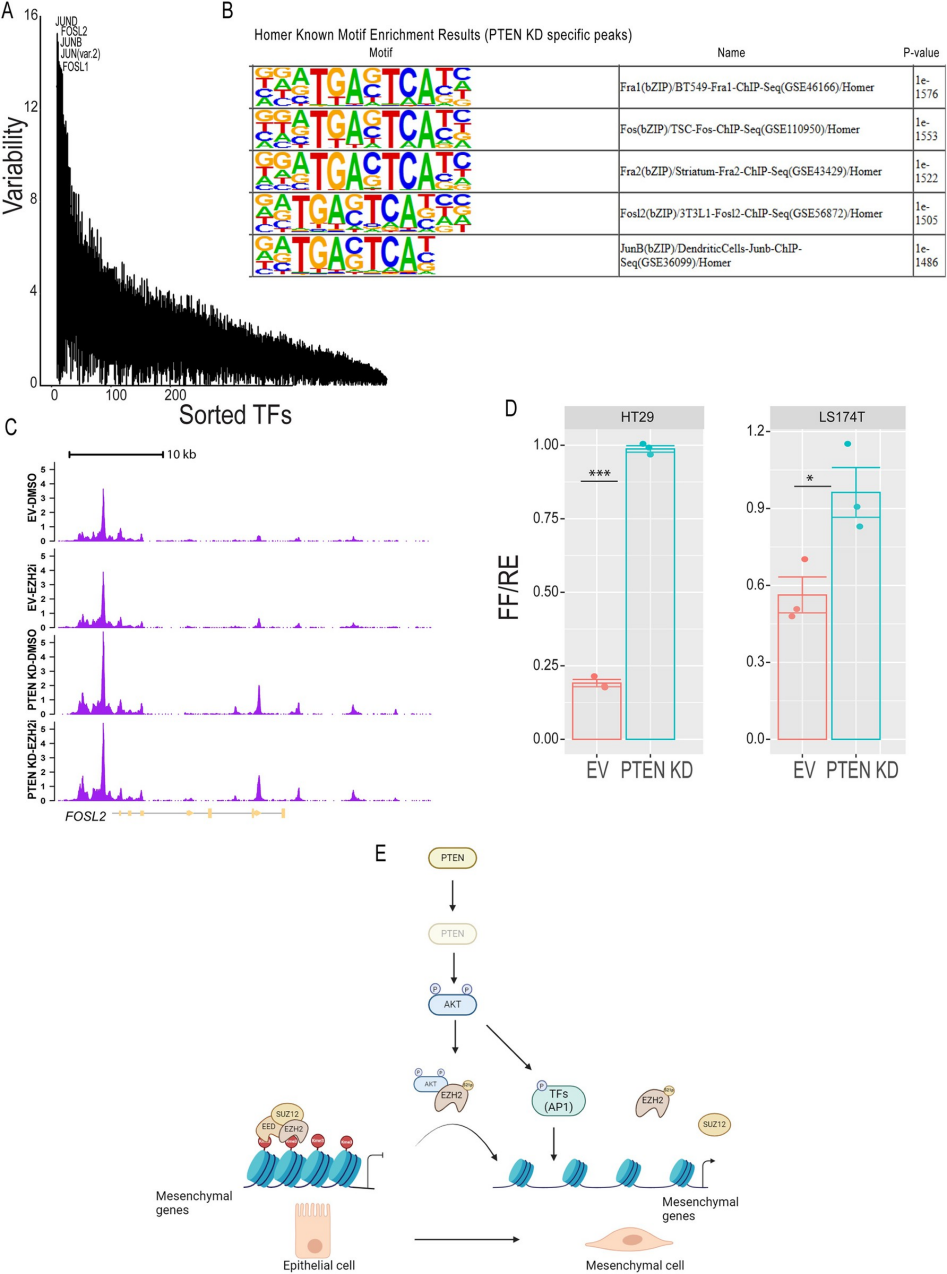
PTEN KD increases AP1 transcriptional activity. **(A)** Motif enrichment analysis for differentially accessible peaks in PTEN knockdown (KD) vs empty vector (EV) HT29 cells from ATAC-seq data using Chromvar. **(B)** Motif enrichment analysis for PTEN KD HT29 specific ATAC-seq peaks determined using Homer. **(C)** ATAC-seq gene tracks for *FOSL2* in EV-DMSO, EV-EZH2i, PTEN KD-DMSO, and PTEN KD-EZH2i samples. the y-axis represents the signal intensity measured as normalized ATAC-seq reads. **(D)** 3X AP1 luciferase assay in HT29 and LS174T cells. PTEN KD and EV cells were plated followed by transfection with both Renella and 3X AP1 luciferase plasmids. Cells were starved in media lacking serum for 24 hours prior to measuring luciferase activity. Luciferase activity was normalized to Renella plasmid as represented by Firefly activity over Renella activity (FR/RE). Results are represented as the mean of 3 biological replicates +SEM. Significance was determined by student t-test. * P ≤ 0.05 and *** P ≤ 0.0001. **(E)** Model for how PTEN KD mediates EMT in epithelial CRC.

The PI3K/AKT pathway has been demonstrated to activate different transcription factors to induce EMT [23, 34]. To gain more insight into DNA-binding transcription factors regulated by PTEN KD, we performed motif enrichment analysis on differentially accessible peaks between EV-DMSO and PTEN KD-DMSO from our ATAC-seq data using two tools, ChromVar and Homer [35, 36]. AP1 subunits, JUND, FOSL2, and JUNB, were the most significant transcription factors in both analyses (Fig 6A and 6B). Additionally, the *FOSL2* gene showed significantly increased accessibility in PTEN KD-DMSO and PTEN KD-EZH2i compared to EV-DMSO and EV-EZH2i ATAC-seq data (Fig 6C). To directly test if PTEN KD alters AP1 transcriptional activity, we performed an AP1 luciferase activity assay. AP1 luciferase activity was significantly higher in PTEN KD compared to EV HT29 and LS174T cells (Fig 6D). Additionally, PTEN KD induced c-JUN phosphorylation at serine 73 (S6 Fig), which has been shown to increase the transcriptional activity of AP1 [37, 38]. Our data suggests that activation of AKT through PTEN KD increases chromatin accessibility at the AP1 subunits, which is accompanied by an increase in AP1 transcriptional activity.

## Discussion

In this study, we demonstrated that PTEN KD drives EMT in CMS3 epithelial CRC. Our data showed that PTEN KD induced an epithelial-to-mesenchymal phenotypic transition in epithelial CRC cells that is associated with increased cell migration. Additionally, PTEN KD increased chromatin accessibility, accompanied by transcriptional reprogramming of the epithelial cells toward mesenchymal CMS4 cells. We also showed that AKT-mediated EZH2 phosphorylation at S21 contributes to the EMT process in epithelial CRC. PTEN KD increased EZH2 interaction with RNAPII and phosphorylation of EZH2 reduced EZH2 interaction with another PRC2 component, SUZ12, leading to a global reduction in H3K27me3.

Consistent with H3K27me3’s role in chromatin compaction [29], our data demonstrated that inhibiting EZH2 activity increased chromatin accessibility in EV HT29 cells. Interestingly, PTEN KD had a much greater effect on chromatin accessibility. Consistent with our ATAC-seq data, PTEN KD also had more of an effect on gene expression compared to inhibiting EZH2 activity alone in HT29 cells. PTEN KD predominantly altered gene expression to mediate EMT in epithelial CMS3 cells. PTEN KD decreased CMS3 probability and increased the expression of EMT-related genes. Additionally, consistent with our previous study in CMS4 SW480 cells [15], inhibiting EZH2 activity further increased CMS4 probability in PTEN KD HT29 cells and pushed cells toward the PDS2 (mesenchymal) classification. CMS and PDS classifiers indicated that EZH2 inhibitor enhances and/or stabilizes PTEN KD-mediated transcriptional reprogramming toward mesenchymal cells. Additionally, unlike the effect of EZH2 inhibitor treatment on cell migration in PTEN KD SW480 [15], EZH2 inhibition did not further enhance cell migration in PTEN KD cells suggesting that the transcriptional changes induced by PTEN KD are sufficient for a migratory phenotypic change.

EZH2 has been shown to bind and repress mesenchymal genes [39, 40]. In our previous work, we showed that inhibiting EZH2 activity alone in mesenchymal CMS4 SW480 cells induced the expression of EMT marker genes [15]. However, inhibiting EZH2 activity alone in epithelial CMS3 HT29 cells had a smaller effect on gene expression and only modestly induced the expression of a few EMT genes. The difference in the response to EZH2 inhibition might be due to the difference in initial cell states of these two cell lines as indicated by their CMS classification. SW480 cells are CMS4, which are characterized by upregulation of EMT marker genes and genes associated with TGFβ signaling at baseline compared to other CMS classes [19]. It was previously shown that treating lung carcinoma or ovarian cancer cells with TGFβ enhanced EZH2 inhibitor-mediated increases in EMT gene expression [39, 40]. Therefore, it is possible that inhibiting EZH2 catalytic activity alone in CMS4 cells is enough to induce EMT gene expression due to baseline TGFβ signaling in CMS4 cells that is not present in CMS3 cells.

Our data further demonstrate that PTEN KD induces a global reduction of H3K27me3 in epithelial CRC. Interestingly, PTEN KD-mediated H3K27me3 reduction is dependent on EZH2 phosphorylation at serine 21 suggesting that it might be regulated by AKT-mediated EZH2 phosphorylation. We have shown previously that AKT interacts with and phosphorylates EZH2 at serine 21 in CRC [15]. Our findings are consistent with previous reports where AKT-mediated EZH2 phosphorylation leads to a global reduction in H3K27me3 [16, 18]. Our data showed that EZH2 PM (S21D) interacted less with SUZ12. Consistent with these findings, EZH2 PM could not restore the H3K27me3 loss induced by EZH2 KD. Our results suggest that phosphorylation of EZH2 via AKT attenuates PRC2 catalytic activity by disrupting PRC2 complex leading to a reduction in H3K27me3. It should be noted that we did not detect a decrease in H3K27me3 in response to PTEN KD in CMS4 cells [15]. Future work is required to understand why disruption of PRC2 occurs in epithelial CMS3 CRC cells and not in CMS4 mesenchymal CRC cells.

Activation of AKT has been shown to mediate EMT via activating different transcription factors [22, 23, 25]. Because PTEN KD both activated AKT and reduced H3K27me3 levels, it suggests that both a global reduction of H3K27me3 and activation of AKT cell signaling are needed to fully increase chromatin accessibility and induce gene expression to promote EMT. Our data showed that activation of AKT activates the transcriptional activity of the AP1 transcription factor. AP1 is a transcription factor family consisting of a diverse group of members including JUN and FOS [41]. Several studies demonstrate the role of AP1 in cancer cell metastasis [26, 42]. For example, Fos-related antigen 1 has been shown to mediate EMT in mammary epithelial cells by inducing the expression of ZEB1 and ZEB2 proteins [26]. Additionally, It was previously demonstrated that AP1 increases chromatin accessibility to promote the expression of glucocorticoid nuclear receptor target genes [43]. Therefore, the increase in AP1 transcriptional activity in response to PTEN KD might explain the global increase in chromatin accessibility in PTEN KD compared to inhibiting EZH2 activity alone in HT29 cells.

Overall, we propose a model where activation of AKT through PTEN KD induces EZH2 phosphorylation at serine 21, which attenuates PRC2 activity, and enhances the transcriptional activity of AP1 to transcriptionally reprogram epithelial CRC cells toward mesenchymal cells (Fig 6E). Our findings suggest that how cells respond to EZH2 inhibition depends on the initial state of the cells (CMS4 versus CMS3) indicating that the role of EZH2 in maintaining cell identity is likely context dependent.

## Limitations of the study

In the present study, we demonstrated that PTEN KD modulates chromatin accessibility to mediate transcriptional reprogramming toward the mesenchymal lineage. Although we used PTEN KD to induce AKT activation, we did not demonstrate that these transcriptional changes are indeed dependent on AKT activation as PTEN KD can act independently from the PI3K/AKT pathway [44]. In future work, we should consider investigating the effect of AKT inhibitors on PTEN KD-mediated EMT. Although we have demonstrated that PTEN KD increases the transcriptional activity of AP1, we did not show if/how the activation of AP1 is involved in the EMT process. Further work is needed to understand the precise mechanism by which AP1 mediates EMT in epithelial CRC. Additionally, we and others have shown that phosphorylation of EZH2 induces EZH2 to interact with transcription factors [15–17]. Therefore, it is possible that EZH2 phosphorylation induces EZH2 to interact with AP1 to regulate AP1 transcriptional activity. Future work is needed to investigate if EZH2 regulates AP1 activity to mediate EMT in epithelial CRC. Future research should also determine how the changes in EMT studied here in two different epithelial CRC cell lines affect metastasis in vivo.

## Materials and methods

### Cell lines

All cell lines were maintained in a humidified atmosphere at 37°C with 5% CO_2_. Our study included HT29, LS174T, and HEK293T cell lines. HT29 cells were cultured in McCoy 5A media (Corning, #10-050-CV). LS174T and HEK293T cells were cultured in DMEM 1X (Corning, #10-013-CV) with 10% FBS (Corning, #35-015-CV) without antibiotics. All cell lines were purchased from the ATCC. HT29 and HEK293T cells were derived from female patients. LS174T cells were derived from a male patient. All cells used in experiments were passaged fewer than 15 times with most being passaged fewer than 10 times. Where indicated, cells were starved in media lacking serum for 24 hours prior to treatment. GSK503 (Sigma, #SML2718) and Tazemetostat (MCE, #HY-13803) were solubilized in DMSO (VWR, #97063–136) prior to treatment. Treatment dosages and durations are defined in the Fig legends.

### Generation of stable knockdown cell lines

For knockdown of EZH2 (Sigma, SHCLNG-NM_004456, #TRCN0000010474), PTEN (Sigma, SHCLNGNM_000314, #TRCN0000002748), and empty vector (EV) TRC2 (Sigma, #SHC201), the lentiviral shRNA knockdown protocol from The RNAi Consortium Broad Institute was used. Briefly, 4 x 10^5^ HEK293T cells were plated on day 1 in DMEM 1X containing 10% FBS. On day 2, cells were transfected with shRNA of interest, EV control, and packaging plasmids. On day 3, the media was replaced with fresh DMEM containing 10% FBS. Approximately 24 hours later, media containing lentiviral particles was collected, and fresh DMEM + 10% FBS was added. The added media was collected 24 hours later and pooled with media harvested on day 4. The pooled media was then filtered using a 0.45 μm filter and concentrated using a Spin-X concentrator (Corning, #431490). To perform the knockdown, concentrated virus plus polybrene was added to experimental cells 24 hours after plating. Cells were treated with puromycin (2 μg/mL) (Sigma-Aldrich, #P8833) after 24 hours to select for knockdown cells.

### Plasmids and transient transfections

HA-EZH2 plasmid mutant constructs were generated in our previous publication [15]. Transient transfection was performed with Lipofectamine 3000 (Invitrogen) per the manufacturer’s protocol.

### Antibodies

For Western blot, anti-RBP1 (CST, #2629, 1:1000), anti-H3K27me3 (CST, #9733, 1:1000), anti-H3K4me3 (CST, #9751, 1:1000), anti-total AKT (CST, #4691,1:1000), anti-Phospho-AKT(Ser473) (CST, #4060, 1:1000), anti-H3 (CST, #4499, 1:1000), anti-GAPDH (CST, #5174, 1:1000), anti-HA (CST, #3724, 1:1000), anti-EZH2 (CST, #5246, 1:1000), and anti-SUZ12 (CST, #3737, 1:1000) antibodies were used.

### Nuclear Immunoprecipitations (IPs)

3.5 x 10^6^ or 1.2 x 10^6^ cells were cultured in 150 mm plates for approximately 72 hours or 96 hours, respectively. Cell pellets were used to perform nuclear extraction using CEBN [10 mmol/L HEPES, pH 7.8, 10 mmol/L KCl, 1.5 mmol/L MgCl2, 0.34 mol/L sucrose, 10% glycerol, 0.2% NP-40, 1X protease inhibitor cocktail (Sigma, #P5726), 1X phosphatase inhibitor (Thermo, #88266), and N-ethylmaleimide (Acros organics, #128-53-0)] and then washed with CEB buffer (CEBN buffer without NP-40) containing all the inhibitors. To extract the nuclear fraction, after washing the cell pellets with CEBN buffer, they were resuspended in modified RIPA (50 mM Tris pH7.5, 150 mM NaCl, 5 mM EDTA, 50 mM NaF, all inhibitors) and sonicated using Bioruptor® Pico (Diagenode). To exclude the possibility of DNA-dependent protein interactions, the nuclear fractions were incubated with spermine (50 mM) and spermidine (15 mM) for 60 minutes on a rotator at 4°C. Positively charged spermines compete proteins off the DNA. The nuclear extract was rotated with antibody and protein A/G magnetic beads overnight. The next day, beads were washed, and proteins were eluted and analyzed by Western blot.

### ATAC-seq

Transposase-accessible chromatin with sequencing (ATAC-seq) was performed using an ATAC-seq kit according to the manufacturer’s protocol (Active motif, #53150). Purified DNA was used to prepare Illumina library using the NEBNext Ultra II DNA Library Prep kit (NEB, #E7645) as per the manufacturer’s protocol, followed by sequencing.

### RNA sequencing

RNA extraction was performed using the RNAeasy mini kit (Qiagen, #74104) as per manufacture’s protocol. Libraries were generated for sequencing using the NEBNext Ultra II DNA Library Prep kit for Illumina (NEB, E7645) as per the manufacturer’s protocol, followed by sequencing.

### Migration assay

7 x 10^4^ HT29 or LS174T cells in serum-free media were plated into transwell in 24-well plates (Corning, #40578) for 48 hours with media containing 10% FBS at the bottom. Transwell inserts were stained using Hema 3 Stat Pack (Thermo Fisher Scientific, #123–869). Migration inserts were randomized before manual quantification and the outer 5% of the inserts were not included during quantification to reduce edge-effect bias. All images were taken on an EVOS FL Auto microscope.

### Colony formation assay

500 cells were plated in a 6-well plate and cultured at 37C. After 15 days, cells were fixed with ice-cold methanol and stained with crystal violet. Crystal violet–stained cells were counted manually, and the images were taken by scanning the plate.

### Immunofluorescence and imaging

HT29 cells (3x10^5^) were cultured on coverslips in a 6-well plate and incubated at 37C, cells were untreated or treated with EZH2 inhibitor (GSK503 (Sigma, #SML2718)) for 72 hours. Cells were starved in media lacking serum for 24 hours prior to staining. Cells were fixed with 4% paraformaldehyde in PBS. Post fixation, cells were permeabilized using 0.5% Triton-X in PBS, blocked with 1% BSA in PBST (PBS + 0.1% Tween-20), incubated with anti-ZO1 (cell signaling technology,#5406, 1:50) in 1% BSA in PBST overnight at 4°C. Then, cells were incubated with Alexa Fluor (AF) -conjugated secondary antibodies (anti-rabbit AF594, CST, #8889) for 2H at room temperature. Images were acquired on a Leica SP8 scanning confocal system 470 with MDi8-inverted microscope with LASX software (Leica Microsystems).

### 3X AP1 Luciferase assay

3xAP1pGL3 (3xAP-1 in pGL3-basic) was a gift from Alexander Dent (Addgene plasmid # 40342; http://n2t.net/addgene:40342; RRID:Addgene_40342) and has three canonical AP-1 binding sites driving luciferase gene expression [45]. Renilla luciferase expression vector, pRL-SV40P, was used to control transfection efficiency. The pRL-SV40P was a gift from Ron Prywes (Addgene plasmid # 27163; http://n2t.net/addgene:27163; RRID:Addgene_27163) [46]. 48 hours after transfection, the cells were lysed, and luciferase activity was measured using the Firefly and Renilla Luciferase Assay Kit (Promega). Relative activity was computed by normalizing the Firefly luciferase activity against the Renilla luciferase.

### RNA isolation and RT-qPCR

Isolation of total RNA from cell pellets was performed using the RNAeasy mini kit (Qiagen, #74104) as per the manufacturer’s protocol. Maxima first strand cDNA synthesis kit (Thermo Fisher, #K1642) for quantitative reverse transcription PCR was used to synthesize cDNA. cDNA was amplified using gene-specific primers and FastStart Essential DNA Green Master (Roche, #06402712001). Cq values of genes of interest were normalized to housekeeping gene *ROHA* expression. qPCR primer sequences are listed in S1 Table.

### Sequencing analysis

Sequencing read quality control for all samples was assessed with FastQC (v0.11.9). For RNA-seq analysis, read alignment was performed using STAR [47] (v2.7.10a) against the hg38 reference genome, raw read counts for genes were obtained with STAR (v2.7.10a), and Deseq2 [48] (v3.24.0) was used for differential expression analysis. Gene Set Enrichment Analysis using the Molecular Signatures Database collections and database were run with clusterProfiler [49] (v1.38.3), and volcanoplot was visualized with EnhancedVolcano [50]. CMS classification and PDS prediction were performed using R packages, CMSclassifier (https://github.com/Sage-Bionetworks/CMSclassifier), and PDSclassifier [28], respectively. For ATAC-seq analysis, read alignment was performed using Bowtie2 [51] (v2.4.2) against the hg38 reference genome, peaks were called with MACS [52] (v2.2.7.1), and differentially accessible peaks were calculated using DiffBind [53]. Heatmaps and Counts Per Million normalized bigWigs were created using deepTools [54] (v3.5.1) bamCoverage, computeMatrix, and plotHeatmap. Gviz [55] (1.34.1) was utilized for creating gene tracks. Motif enrichment analyses were performed using ChromVar and HOMER [36, 36]. Metascape pathway analysis was performed for pathway analysis [30].

### Quantification and statistical analysis

Relative H3K27me3 band intensity in Western blots were measured by imageJ and normalized to the housekeeping gene GAPDH. Results are represented as mean +/- SEM unless otherwise stated. Significance was determined by one-way ANOVA with the Tukey multiple comparisons test using RStudio otherwise stated. For all western blots and IPs, data presented is representative of at least two independent biological replicates. For all Figs, * P ≤ 0.05, ** P ≤ 0.01, *** P ≤ 0.001, **** P ≤ 0.0001. All significant comparisons are shown. Statistical details for each experiment are included in the Figs and Fig legends.

## Supporting information

S1 Fig PTEN KD increases the expression of a subset of EMT genes in epithelial CRC cells. **A)** Gene expression of the indicated genes by RT-qPCR in HT29 and LS174T cells treated with DMSO or 2 μM EZH2 inhibitor (EZH2i, GSK-503) for 72 h. The relative expression levels of indicated genes were measured using the Delta Delta Cq method. Cq values were normalized to the housekeeping gene *RHOA* expression and then to EV DMSO cells. Cells were starved in media lacking serum for 24 hours prior to RNA extraction. Results are represented as the mean of 3 biological replicates +/- SEM. (**B)** Crystal violet–stained colonies formed in EV and PTEN KD HT29 and LS174T. Cells were plated in a 6-well plate and cultured at 37°C. After 15 days, cells were stained with crystal violet. Crystal violet–stained cells were counted manually, and the images were taken by scanning the plate. (**C)** Quantification of number of colonies per well from experiment in B. Results are represented as the mean of 3 biological replicates +/- SEM. Significance was determined by one-way ANOVA with the Tukey multiple comparisons test. All significant comparisons are shown. * P ≤ 0.05, ** P ≤ 0.01, *** P ≤ 0.001, **** P ≤ 0.0001.
(TIF)

S2 Fig EZH2 inhibition has a minor effect on gene transcription. **(A)** Volcanoplot for the effect of EZH2i on PTEN KD-regulated genes in HT29 cells. Dashed lines represent Log2 Fold change > |1| and p-adj value <0.05. Red dots indicate genes significantly up or down regulated compared to PTEN-KD-EZH2i with Log2 Fold change > |1|, blue dots indicate genes Log2 Fold change < |1|. Black dots indicate nonsignificant gene expression. **(B)** Volcanoplot for the DEGs in EV-EZH2i versus EV-DMSO in HT29 cells. Dashed lines represent Log2 Fold change > |1| and p-adj value <0.05. Red dots indicate genes significantly up or down regulated compared to EV-EZH2i with Log2 Fold change > |1|, blue dots indicate genes Log2 Fold change < |1|. Black dots indicate nonsignificant gene expression**. (C)** Bar plot for gene ontology generated by Metascape for PTEN KD-upregulated genes in PTEN KD versus EV-DMSO HT29 cells. **(D)** Bar plot for gene ontology generated by Metascape for PTEN KD-EZH2i-upregulated genes in PTEN KD-EZH2i versus EV-DMSO HT29 cells. **(E)** Bar plot for gene ontology generated by Metascape for PTEN KD-downregulated genes in PTEN KD-DMSO versus EV-DMSO HT29 cells. **(F)** Bar plot for gene ontology generated by Metascape for PTEN KD-EZH2i-downregulated genes in PTEN KD-EZH2i versus EV-DMSO HT29 cells.
(TIF)

S3 Fig PTEN KD increases chromatin accessibility. **(A)** Western blots of total cell lysate prepared from EV and PTEN KD HT29 cells. Cells were starved in media lacking serum for 24 hours prior to protein extraction. **(B)** Metagenomic heatmap for second replicate of ATAC-seq prepared from EV and PTEN KD HT29 cells treated with DMSO or 2 μM EZH2 inhibitor (EZH2i, GSK-503) for 72 hrs. The heatmap displays the chromatin accessibility profiles of the indicated samples. Peaks were combined across all samples. The color legend represents the magnitude of each peak. The higher the number the higher the chromatin accessibility. **(C)** Dotplot for pathways for genes with increased chromatin accessibility in EV-DMSO versus PTEN KD-DMSO HT29 cells.
(TIF)

S4 Fig PTEN KD increases chromatin accessibility at EMT genes. **A)** Pearson’s correlation analysis between differentially accessible genes (DAGs) in ATAC-seq data (log 2FC>0.5; p < 0.05) and differentially expressed genes (DEGs) in RNA-seq data (log 2FC>0.5; p < 0.05) in PTEN KD-DMSO versus EV-DMSO in HT29 cells. **(B)** Vennplot for differentially accessible genes (DAGs) in ATAC-seq data and differentially expressed genes (DEGs) in RNA-seq data in PTEN KD-DMSO versus EV-DMSO HT29 cells. **(C)** ATAC-seq gene tracks for indicated genes in EV-DMSO, EV-EZH2i, PTEN KD-DMSO, and PTEN KD-EZH2i HT29 cells. the y-axis represents the signal intensity measured as normalized ATAC-seq reads **(D)** RT-qPCR for indicated genes in EV and PTEN KD HT29 cells. Cells were starved in media lacking serum for 24 hours prior to RNA extraction. The relative expression levels of indicated genes were measured using the Delta Delta Cq method. Cq values were normalized to the housekeeping gene *RHOA* expression and then to EV. Results are represented as the mean of 3 biological replicates +SEM. Significance was determined by student t-test. ** P ≤ 0.01, ns = non-significant. **(E)** Gene ontology for common genes in (B) generated by Metascape.
(TIF)

S5 Fig Phosphorylation of EZH2 disrupts PRC2 integrity. **(A)** SUZ12 IP in LS174T cells. LS174T cells were plated followed by transfection with HA-EZH2 WT or HA-EZH2 PM followed by nuclear protein isolation. Nuclear protein lysates were used for the IP. IP with beads serves as a negative control. Input is nuclear lysates used for IP. **(B)** Western blots of total cell lysates prepared from EZH2 KD LS174T cells transfected with HA-EZH2 WT and HA-EZH2-PM. EZH2 KD LS174T were plated followed by mock transfection or transfection with HA-EZH2 WT or HA-EZH2-PN. **(C)** EZH2 IP performed using nuclear lysates prepared from EV and PTEN KD LS174T. Cells were starved in media lacking serum for 24 hours prior to nuclear protein extraction. IP with beads serves as a negative control. Input is nuclear lysates used for IP.
(TIF)

S6 Fig PTEN KD increases c-JUN phosphorylation. Western blots of total cell lysate prepared from PTEN KD and EV HT29 cells. Cells were starved in media lacking serum for 24 hours prior to preparing cell lysate.
(TIF)

S1 Table Sequences of primers used RT-qPCR. (DOCX)
