# Supplementary figures and images for: PTEN depletion reduces H3K27me3 levels to promote epithelial-to-mesenchymal transition in epithelial colorectal cancer cells
Source: PLoS One. 2024 Nov 19;19(11):e0313769. doi: 10.1371/journal.pone.0313769 (PMC11575820; doi:10.1371/journal.pone.0313769)

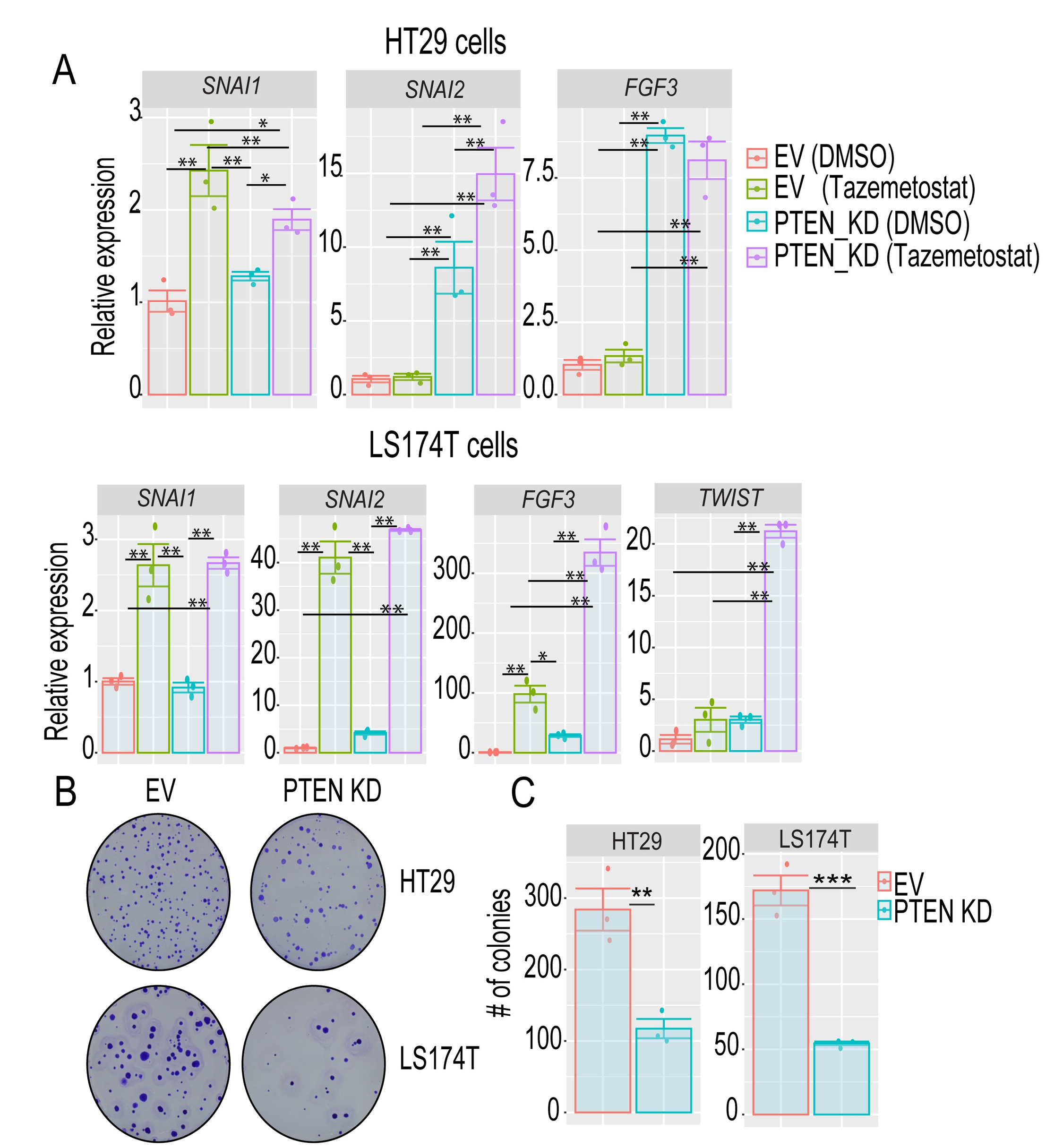

Supplement: S1 Fig — A) Gene expression of the indicated genes by RT-qPCR in HT29 and LS174T cells treated with DMSO or 2 μM EZH2 inhibitor (EZH2i, GSK-503) for 72 h. The relative expression levels of indicated genes were measured using the Delta Delta Cq method. Cq values were normalized to the housekeeping gene RHOA expression and then to EV DMSO cells. Cells were starved in media lacking serum for 24 hours prior to RNA extraction. Results are represented as the mean of 3 biological replicates +/- SEM. (B) Crystal violet–stained colonies formed in EV and PTEN KD HT29 and LS174T. Cells were plated in a 6-well plate and cultured at 37°C. After 15 days, cells were stained with crystal violet. Crystal violet–stained cells were counted manually, and the images were taken by scanning the plate. (C) Quantification of number of colonies per well from experiment in B. Results are represented as the mean of 3 biological replicates +/- SEM. Significance was determined by one-way ANOVA with the Tukey multiple comparisons test. All significant comparisons are shown. * P ≤ 0.05, ** P ≤ 0.01, *** P ≤ 0.001, **** P ≤ 0.0001. (TIF) [file pone.0313769.s001.tif]

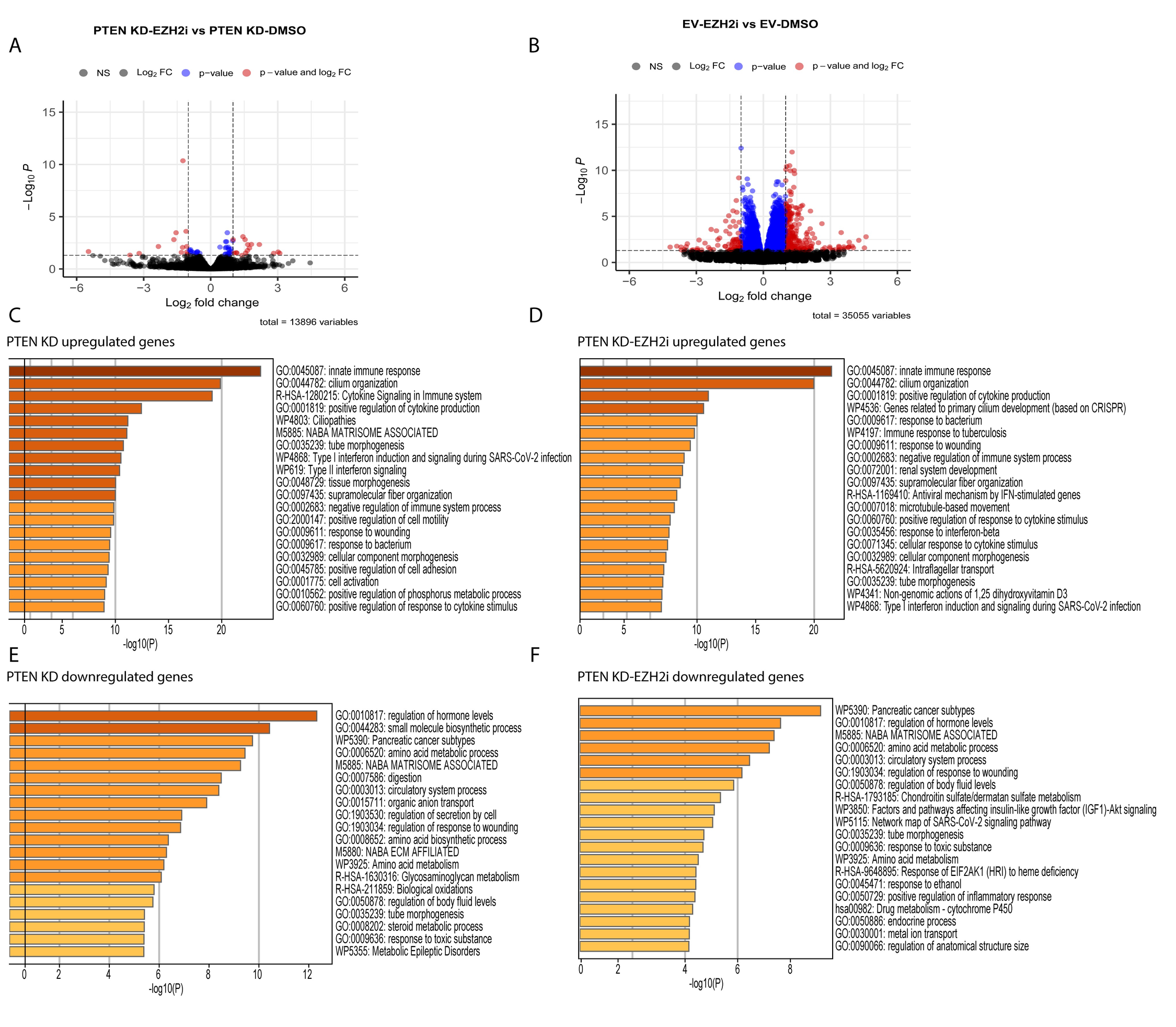

Supplement: S2 Fig — (A) Volcanoplot for the effect of EZH2i on PTEN KD-regulated genes in HT29 cells. Dashed lines represent Log2 Fold change > |1| and p-adj value <0.05. Red dots indicate genes significantly up or down regulated compared to PTEN-KD-EZH2i with Log2 Fold change > |1|, blue dots indicate genes Log2 Fold change < |1|. Black dots indicate nonsignificant gene expression. (B) Volcanoplot for the DEGs in EV-EZH2i versus EV-DMSO in HT29 cells. Dashed lines represent Log2 Fold change > |1| and p-adj value <0.05. Red dots indicate genes significantly up or down regulated compared to EV-EZH2i with Log2 Fold change > |1|, blue dots indicate genes Log2 Fold change < |1|. Black dots indicate nonsignificant gene expression. (C) Bar plot for gene ontology generated by Metascape for PTEN KD-upregulated genes in PTEN KD versus EV-DMSO HT29 cells. (D) Bar plot for gene ontology generated by Metascape for PTEN KD-EZH2i-upregulated genes in PTEN KD-EZH2i versus EV-DMSO HT29 cells. (E) Bar plot for gene ontology generated by Metascape for PTEN KD-downregulated genes in PTEN KD-DMSO versus EV-DMSO HT29 cells. (F) Bar plot for gene ontology generated by Metascape for PTEN KD-EZH2i-downregulated genes in PTEN KD-EZH2i versus EV-DMSO HT29 cells. (TIF) [file pone.0313769.s002.tif]

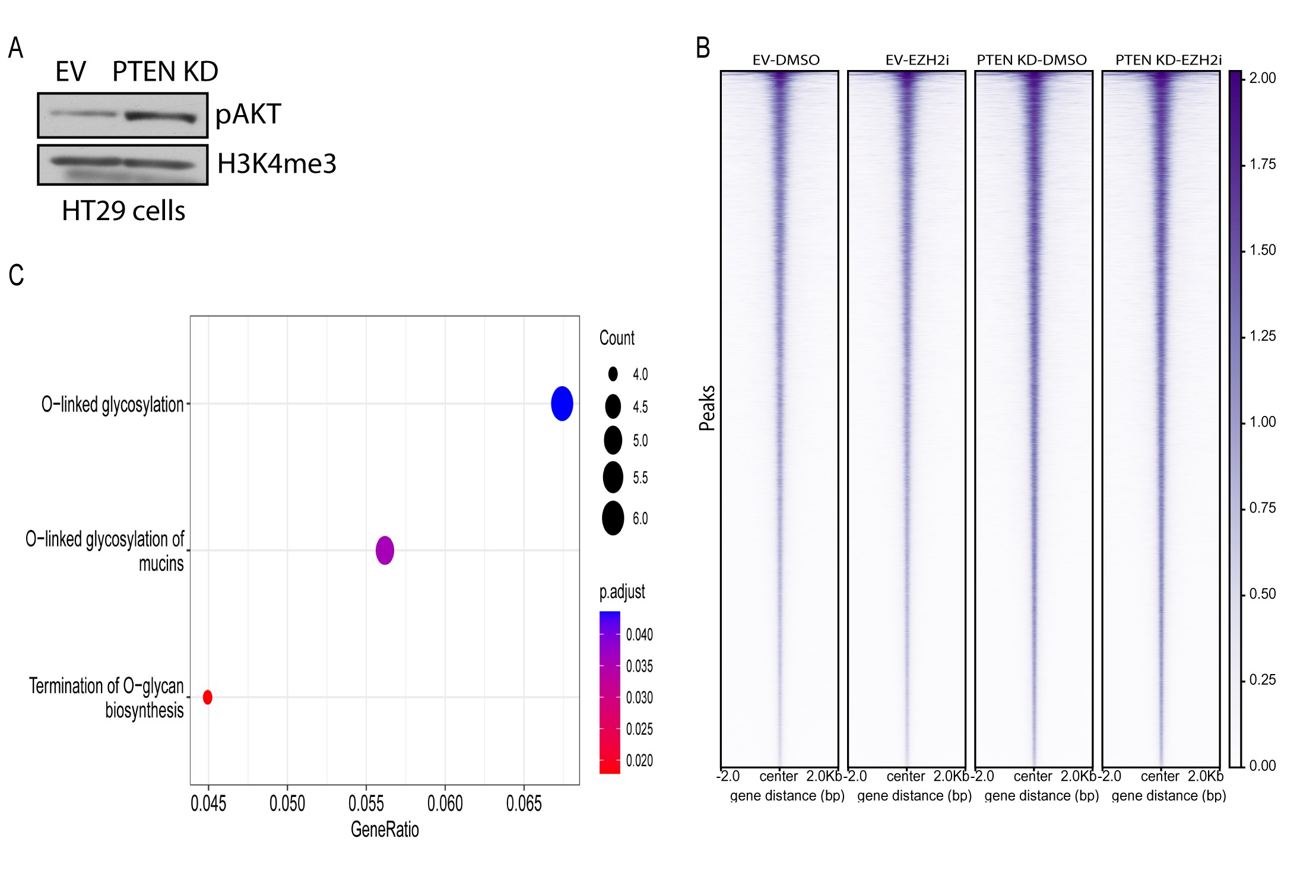

Supplement: S3 Fig — (A) Western blots of total cell lysate prepared from EV and PTEN KD HT29 cells. Cells were starved in media lacking serum for 24 hours prior to protein extraction. (B) Metagenomic heatmap for second replicate of ATAC-seq prepared from EV and PTEN KD HT29 cells treated with DMSO or 2 μM EZH2 inhibitor (EZH2i, GSK-503) for 72 hrs. The heatmap displays the chromatin accessibility profiles of the indicated samples. Peaks were combined across all samples. The color legend represents the magnitude of each peak. The higher the number the higher the chromatin accessibility. (C) Dotplot for pathways for genes with increased chromatin accessibility in EV-DMSO versus PTEN KD-DMSO HT29 cells. (TIF) [file pone.0313769.s003.tif]

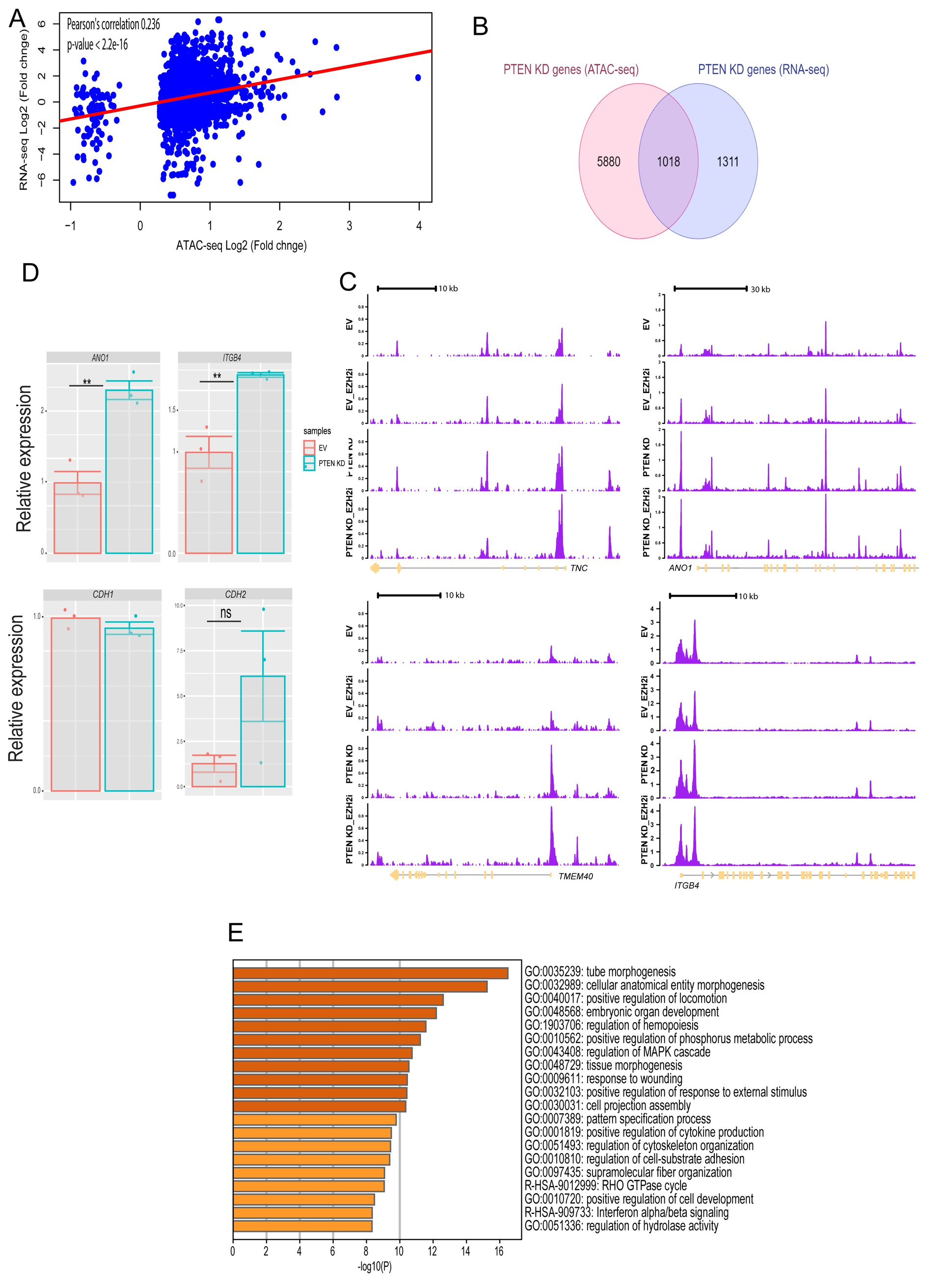

Supplement: S4 Fig — A) Pearson’s correlation analysis between differentially accessible genes (DAGs) in ATAC-seq data (log 2FC>0.5; p < 0.05) and differentially expressed genes (DEGs) in RNA-seq data (log 2FC>0.5; p < 0.05) in PTEN KD-DMSO versus EV-DMSO in HT29 cells. (B) Vennplot for differentially accessible genes (DAGs) in ATAC-seq data and differentially expressed genes (DEGs) in RNA-seq data in PTEN KD-DMSO versus EV-DMSO HT29 cells. (C) ATAC-seq gene tracks for indicated genes in EV-DMSO, EV-EZH2i, PTEN KD-DMSO, and PTEN KD-EZH2i HT29 cells. the y-axis represents the signal intensity measured as normalized ATAC-seq reads (D) RT-qPCR for indicated genes in EV and PTEN KD HT29 cells. Cells were starved in media lacking serum for 24 hours prior to RNA extraction. The relative expression levels of indicated genes were measured using the Delta Delta Cq method. Cq values were normalized to the housekeeping gene RHOA expression and then to EV. Results are represented as the mean of 3 biological replicates +SEM. Significance was determined by student t-test. ** P ≤ 0.01, ns = non-significant. (E) Gene ontology for common genes in (B) generated by Metascape. (TIF) [file pone.0313769.s004.tif]

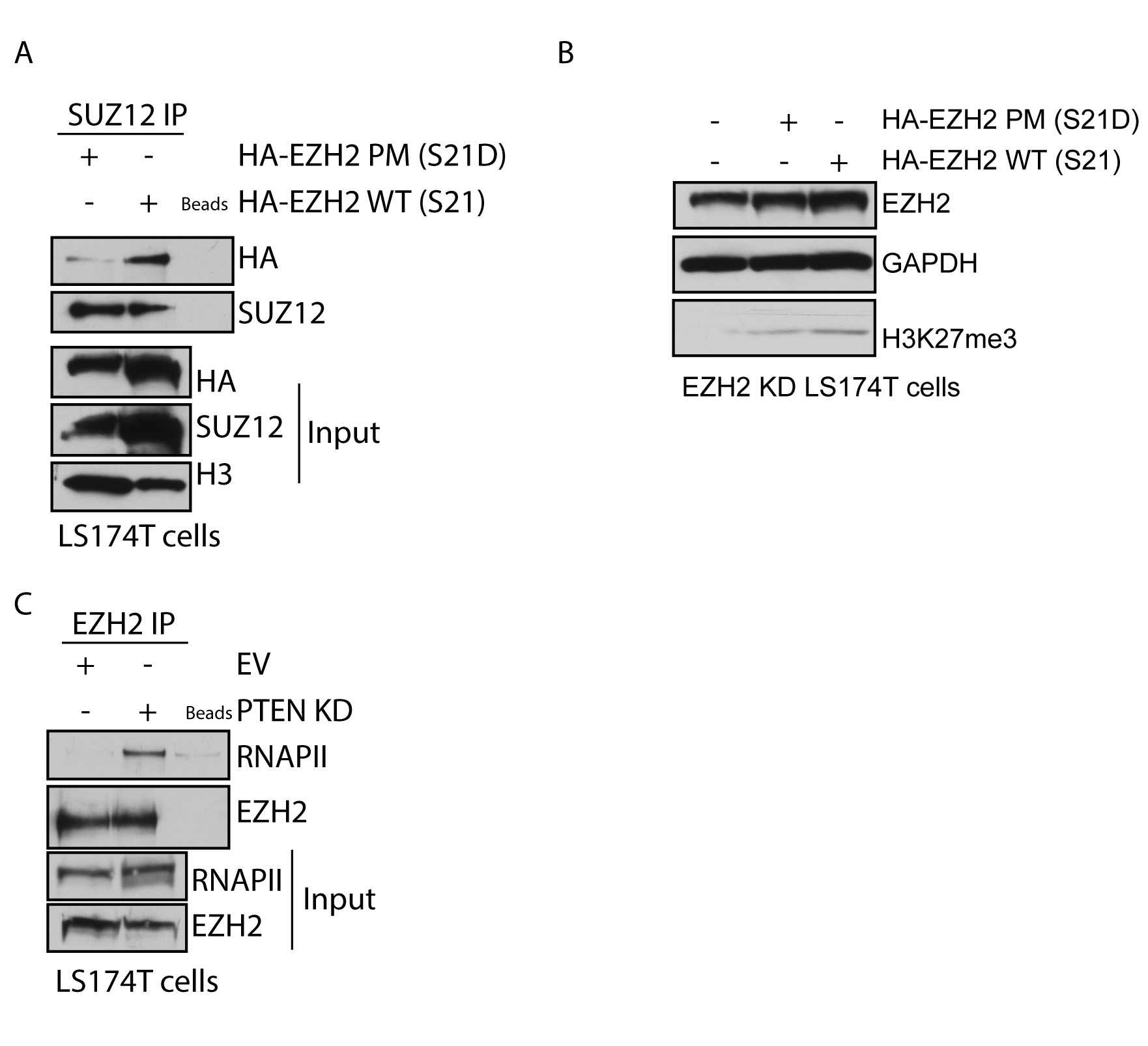

Supplement: S5 Fig — (A) SUZ12 IP in LS174T cells. LS174T cells were plated followed by transfection with HA-EZH2 WT or HA-EZH2 PM followed by nuclear protein isolation. Nuclear protein lysates were used for the IP. IP with beads serves as a negative control. Input is nuclear lysates used for IP. (B) Western blots of total cell lysates prepared from EZH2 KD LS174T cells transfected with HA-EZH2 WT and HA-EZH2-PM. EZH2 KD LS174T were plated followed by mock transfection or transfection with HA-EZH2 WT or HA-EZH2-PN. (C) EZH2 IP performed using nuclear lysates prepared from EV and PTEN KD LS174T. Cells were starved in media lacking serum for 24 hours prior to nuclear protein extraction. IP with beads serves as a negative control. Input is nuclear lysates used for IP. (TIF) [file pone.0313769.s005.tif]

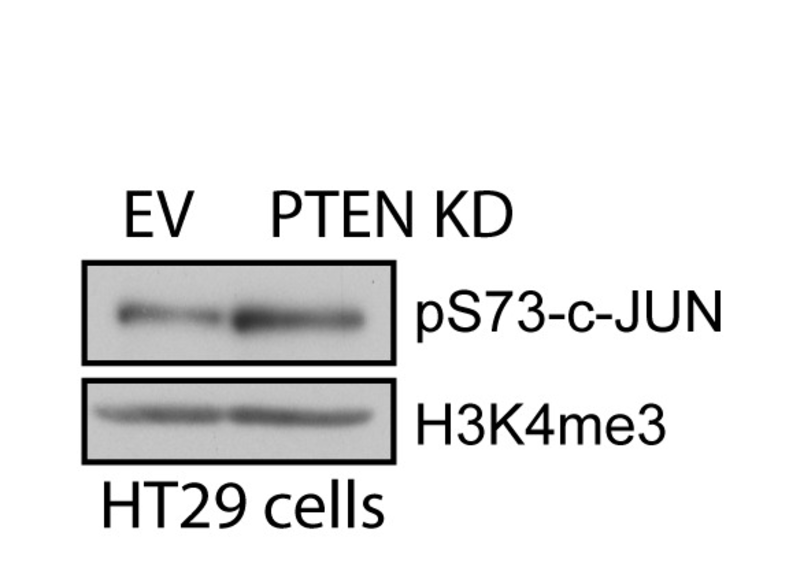

Supplement: S6 Fig — Western blots of total cell lysate prepared from PTEN KD and EV HT29 cells. Cells were starved in media lacking serum for 24 hours prior to preparing cell lysate. (TIF) [file pone.0313769.s006.tif]
