# Supplementary material for: PTEN depletion reduces H3K27me3 levels to promote epithelial-to-mesenchymal transition in epithelial colorectal cancer cells
Source: PLoS One. 2024 Nov 19;19(11):e0313769. doi: 10.1371/journal.pone.0313769 (PMC11575820; doi:10.1371/journal.pone.0313769)
Supplement: S1 Table — (DOCX) [file pone.0313769.s007.docx]

| **Primers** | **Sequences** |
| --- | --- |
| *ITGB4* forward primer | GAGGTAGGTCCAGGACGGG |
| *ITGB4* reverse primer | GTTTGCCAAGGTCCCAGAGA |
| *ANO1* forward primer | CTGTCTTCATGGCCCTCTGG |
| *ANO1* reverse primer | TGGCTTCGTATTCAGCTCTAGG |
| *SNAI1* forward primer | CTAGGCCCTGGCTGCTACA |
| *SNAI1* reverse primer | TGGCACTGGTACTTCTTGACA |
| *SNAI2* forward primer | CTCATCTTTGGGGCGAGTGA |
| *SNAI2* reverse primer | CAATGGCATGGGGGTCTGAA |
| *FGF3* forward primer | CAAGAGGGGACGACTCTATGC |
| *FGF3* reverse primer | GGCCCCAGGCGTACTAGA |
| *RHOA* forward primer | CGTTAGTCCACGGTCTGGTC |
| *RHOA* reverse primer | ACCAGTTTCTTCCGGATGGC |
| *CDH1* forward primer | GCCCTGCCAATCCCGATGAAA |
| *CDH1* reverse primer | GGGGTCAGTATCAGCCGCT |
| *CDH2* forward primer | AGCCAACCTTAACTGAGGAGT |
| *CDH2* reverse primer | GGCAAGTTGATTGGAGGGATG |

**S1 Table**. **Sequences of primers used RT-qPCR**
